# Lignans from the Roots and Rhizomes of *Dysosma versipellis* and Their Cytotoxic Activities

**DOI:** 10.3390/molecules28072909

**Published:** 2023-03-24

**Authors:** Yanjun Sun, Haojie Wang, Ruijie Han, Hongyun Bai, Meng Li, Junmin Wang, Weisheng Feng

**Affiliations:** 1 Collaborative Innovation Center for Respiratory Disease Diagnosis and Treatment & Chinese Medicine, Development of Henan Province, Henan University of Chinese Medicine, Zhengzhou 450046, China; 2 School of Pharmacy, Henan University of Chinese Medicine, Zhengzhou 450046, China; 3 Henan Research Center for Special Processing Technology of Chinese Medicine, Zhengzhou 450046, China

**Keywords:** *Dysosma versipellis*, dibenzyltyrolactone, arylnaphthalide, cytotoxic

## Abstract

One new dibenzyltyrolactone lignan dysoslignan A (**1**), three new arylnaphthalide lignans dysoslignan B–C (**2**–**4**), along with fourteen known metabolites (**5**–**18**), were isolated from the roots and rhizomes of *Dysosma versipellis*. Their structures and stereochemistry were determined from analysis of NMR spectroscopic and circular dichroism (CD) data. Compound **2** represents the first report of naturally occurring arylnaphthalide lignan triglycoside. The cytotoxic activities of all isolated compounds were evaluated against A-549 and SMMC-7721 cell lines. Compounds **7**–**10** and **14**–**16** were more toxic than cisplatin in two tumor cell lines. This investigation clarifies the potential effective substance basis of *D. versipellis* in tumor treatment.

## 1. Introduction

Arylnaphthalide lignans have received much attention due to their potent antiviral, antineoplastic, anti-inflammatory, and immunosuppressive properties [1]. The representative effective component (such as podophyllotoxin) has been the subject of extensive research on new antiviral and antineoplastic drugs. Podophyllotoxin tincture is used clinically to treat condyloma acuminatum. Podophyllotoxin derivatives, for instance etoposide and teniposide, are the frontline chemotherapeutic drugs against various cancers. Since remote times, plants containing podophyllotoxin and its analogues have been used by diverse nationalities as laxatives and for the treatment of gonorrhea, tuberculosis, menstrual disorders, psoriasis, dropsy, cough, syphilis and venereal warts [2,3]. So a medicinal plant rich in arylnaphthalide lignans is an important source of natural anticancer agents.

**Figure 1:**
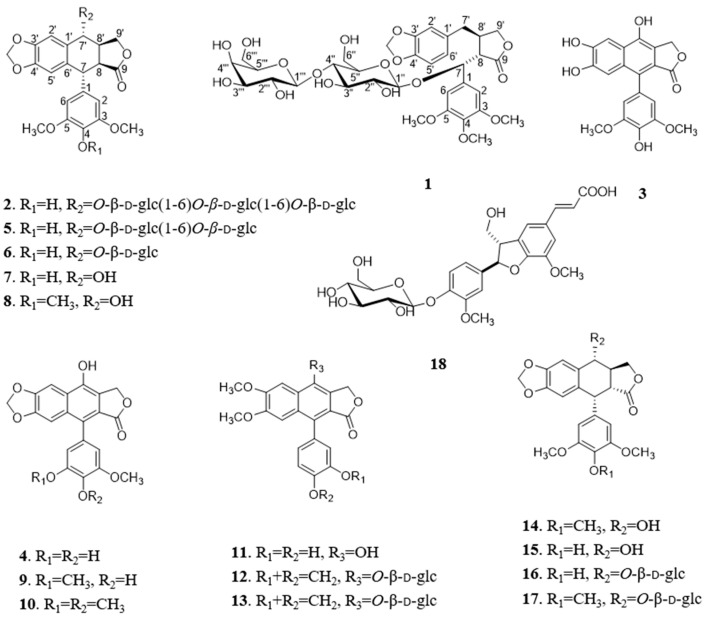
Chemical structures of compounds **1**–**18.**

*Dysosma versipellis* (Hance) M. Cheng ex Ying, belonging to the family of Berberidaceae, is widely distributed in the central/south regions of China [4]. As an important medicinal plant, it has been described in *Shennong’s Herbal Classic*. Its dried roots and rhizomes (called “Bajiaolian” in Chinese) are mainly used for the treatment of parotitis [4], sore throat, snake bite, fall injury [5], epidemic encephalitis B [6], epidemic hemorrhagic fever, condyloma accuminata, and esophagus and breast carcinoma [7]. Previous phytochemical and pharmacological investigations revealed that *D. versipellis* is particularly rich in arylnaphthalide lignans and biflavonoids, and has attracted wide attention due to their cytotoxic and neuraminidase and acetylcholinesterase inhibitory properties [8,9,10,11]. In our search for cytotoxic natural products, one dibenzyltyrolactone lignan dysoslignan A (**1**), three new arylnaphthalide lignans dysoslignans B–C (**2**–**4**), along with fourteen known metabolites (**5**–**18**), were isolated from the roots and rhizomes of *D. versipellis* (Figure 1). Reported herein are their detailed isolation, structure elucidation, and cytotoxic activity.

## 2. Results and Discussion

The 95% EtOH and 50% EtOH extract of the roots and rhizomes of *D. versipellis* were adsorbed by silicious earth, and then fractioned by CH_2_Cl_2_, EtOAc, and MeOH, respectively. The MeOH extract was isolated and purified by repeated column chromatography, allowing the isolation of one new dibenzyltyrolactone lignan dysoslignan A (**1**), three new arylnaphthalide lignans dysoslignan B–C (**2**–**4**), along with fourteen known metabolites (**5**–**18**). By comparing their physical and spectroscopic data with literature values, the known metabolites were identified as sinolignan B (**5**) [12], 4-demethylpicropodophyllotoxin 7′-*O*-β-d-glucopyranoside (**6**) [13], 4-demethylpicropodophyllotoxin (**7**) [13], picropodophyllotoxin (**8**) [13], 4-demethyldehydropodophyllotoxin (**9**) [12], dehydropodophyllotoxin (**10**) [12], taiwanin H (**11**) [14], cleistanthin B (**12**) [15], arabelline (**13**) [16], podophyllotoxin (**14**) [12], 4-demethylpodophyllotoxin (**15**) [12], 4-demethylpodophyllotoxin 7′-*O*-β-d-glucopyranoside (**16**) [12], podophyllotoxin 7′-*O*-β-d-glucopyranoside (**17**) [12], and aegineoside (**18**) [17].

**Figure 2:**
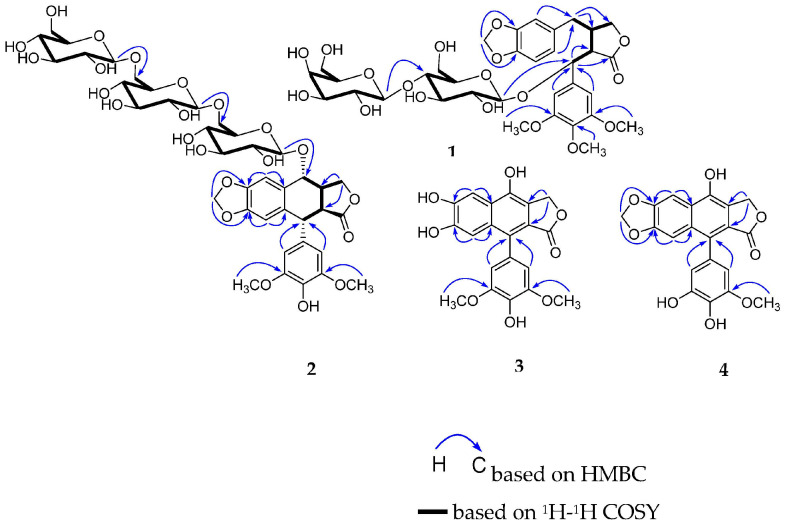
Key ^1^H-^1^H COSY and HMBC correlations of compounds **1**–**4.**

Compound **1** was obtained as a white amorphous powder and its molecular formula was determined as C_34_H_44_O_18_ on the basis of its HR-ESI-MS (*m*/*z* 763.2419 [M + Na]^+^, calcd for 763.2425). The ^1^H NMR spectrum (Table 1 and Figure S1) showed three methoxy groups at δ 3.73 (6H, s), 3.63 (3H, s); one 1,3,4-tri-substituted benzene ring at δ 6.40 (1H, d, *J* = 1.0 Hz), 6.64 (1H, d, *J* = 7.9 Hz), 6.35 (1H, dd, *J* = 7.9, 1.0 Hz); one 1,3,4,5-tetra-substituted benzene ring at δ 6.76 (2H, s); and one methylenedioxy group at δ 5.92 (1H, s), 5.94 (1H, s). The ^13^C NMR spectrum (Table 1 and Figure S2) exhibited one carbonyl group at δ 176.7; twelve aromatic carbons and five aliphatic carbons at δ 76.8, 51.7, 38.6, 36.0, 72.1; as well as three methoxy groups at δ 55.5 (×2), 59.9; one methylenedioxy group at δ 100.7; one set of glucopyranosyl group at δ 99.8, 73.5, 76.5, 81.0, 76.3, 61.0; and one set of galactopyranosyl group at δ 103.2, 73.3, 74.6, 70.8, 74.7, 61.1. The aglycone was identified as poporhizol by comparison of its NMR and ECD data with those reported in the literature [18], combined with data observed in the HSQC, HMBC, DEPT, ^1^H-^1^H COSY, NOESY, and HR-ESI-MS spectra (Figures S3–S9). The ^13^C NMR chemical shifts δ 99.8, 103.2, and spin-spin coupling constants (7.8, 7.9 Hz) of two anomeric protons allowed the identification of β-glucopyranosyl and β-galactopyranosyl moieties. The absolute configurations of glucose and galactose were determined by a microhydrolysis method and HPLC analysis [19]. The HMBC cross peaks (Figure 2) of the anomeric proton at δ 4.14 (1H, d, *J* = 7.8 Hz, H-1″) with C-7 (δ 76.8) and the other anomeric proton at δ 4.26 (1H, d, *J* = 7.9 Hz, H-1‴) with C-4″ (δ 81.0), respectively, indicated that the sugar sequence was β-d-glucopyranosyl-(1→4)-β-d-galactopyranosyl group and was attached at C-7 of the aglycone.

Establishment of the relative configuration was based on the chemical shift of H-9′ and NOESY experiment (Figure S7). NOE correlation of H-7 (δ 5.23) with H-8′ (δ 2.85) indicated that the relationship for H-8/H-8′ was trans. This was also supported by the Δδ Hα-9′-Hβ-9′ value of 0.35 (this value ≥ 0.2 for trans, and ≈ 0 for cis) [20]. The ECD spectrum of **1** (Figure S8) was in good agreement with the ECD spectrum of the 7R,8S,8′R-isomer cleistonkiside B [20]. So the 7R, 8S, and 8′R-configurations were assigned for **1**. Thus, compound **1** was identified as poporhizol 7-*O*-β-d-glucopyranosyl-(1→4)-β-d-galactopyranoside, and named dysoslignan A.

Compound **2** was obtained as a white amorphous powder and its molecular formula was determined as C_39_H_50_O_23_ on the basis of its HR-ESI-MS (*m*/*z* 909.2637 [M + Na]^+^, calcd for 909.2641). The ^1^H NMR spectrum (Table 1 and Figure S10) showed two methoxy groups at δ 3.73 (6H, s), four aromatic protons at δ 7.23 (1H, s), 5.95 (1H, s), 6.57 (2H, s); and one methylenedioxy group at δ 5.94 (1H, s), 5.86 (1H, s). The ^13^C NMR spectrum (Table 1 and Figure S11) exhibited one carbonyl group at δ 178.1, twelve aromatic carbons and five aliphatic carbons, as well as two methoxy groups at δ 56.2 (×2), one methylenedioxy group at δ 100.9, and three sets of glucopyranosyl groups at δ 103.6, 73.9, 77.0, 70.4, 76.9, 68.4, 103.4, 73.6, 76.8, 70.1, 76.8, 68.3, 103.2, 73.5, 76.4, 69.8, 75.0, 61.1. The aglycone was identified as picropodophyllotoxin by comparison of its NMR data with those reported in the literature [21], combined with data observed in the HSQC, HMBC, DEPT, ^1^H-^1^H COSY, NOESY, and HR-ESI-MS spectra (Figures S12–S18). The ^13^C NMR chemical shifts δ 103.6, 103.4, 103.2 and spin-spin coupling constants (7.4, 8.3, 8.0 Hz) of three anomeric protons allowed the identification of three β-glucopyranosyl moieties. The absolute configuration of glucose was determined by the same method as compound **2**. The HMBC cross peaks (Figure 2) of the anomeric proton at δ 4.51 (1H, d, *J* = 7.4 Hz, H-1″) with C-7′ (δ 76.6), and the other two anomeric protons at δ 4.12 (1H, d, *J* = 8.3 Hz, H-1‴) and δ 4.10 (1H, d, *J* = 8.0 Hz, H-1⁗) with C-6″ (δ 68.4) and C-6‴ (δ 68.3), respectively, indicated that the sugar sequence was β-d-glucopyranosyl-(1→6)-β-d-glucopyranosyl (1→6)-β-d-glucopyranosyl group and was attached at C-7′ of the aglycone.

Establishment of the relative configuration was based on the chemical shift of C-9, the ^1^H coupling constants (*J* values) and NOESY experiment (Figure S16). For a cis-orientation of lactone at C-8′ and C-8, the signal of C-9 was at around δ 178.0 ppm, while for a trans-orientation, the signal of C-9 upfield shifted to around δ 175.0 ppm [21]. According to a signal of C-9 at δ 178.1, the orientation of H-8′/H-8 of compound **2** was determined to be *cis*. The *J*_H-7/H-8_ (8.1 Hz) and *J*_H-7_^′^_/H-8_^′^ (10.0 Hz) values indicated the trans-forms of H-7/H-8 and H-7′/H-8′. The NOE correlation of H-7/H-7′ and H-8/H-8′ also supported the relative configuration of 7,8-trans-7′,8′-trans-8,8′-cis. Studies on the ECD curves of 7-aryltetralin lignans showed that all 7β (*S*)-aryl compounds gave negative Cotton effects at around 280–290 nm, while all 7α (*R*)-aryl compounds gave a positive Cotton effect [12]. The ECD spectrum (Figure S17) of compound **2** exhibited a positive Cotton effect at 290 nm. Consequently, the absolute configuration of C-7 was determined to be *R*. Thus, compound **2** was established as 4-demethylpicropodophyllotoxin 7′-*O*-β-d-glucopyranosyl-(1→6)-β-d-glucopyranosyl-(1→6)-β-d-glucopyranoside, and named dysoslignan B.

Compound **3** was obtained as a white amorphous powder and possessed a molecular formula C_20_H_16_O_8_, as revealed by its HR-ESI-MS analysis (*m*/*z* 407.0737 [M + Na]^+^, calcd for 407.0743). The ^1^H NMR spectrum (Table 2 and Figure S19) showed two methoxy group at δ 3.72 (6H, s); four aromatic protons at δ 7.54 (1H, s), 7.03 (1H, s), 6.48 (2H, s); and one methylene group at δ 5.30 (2H, s). The ^13^C NMR spectrum (Table 2 and Figure S20) revealed a skeleton of arylnaphthalide lactone lignan including one carbonyl group at δ 169.9, sixteen aromatic carbons and one aliphatic carbon at δ 66.5, as well as two methoxy groups at δ 56.0 (×2). A careful comparison of the NMR spectra of **3** with 4-demethyl-dehydropodophyllotoxin, combined with data observed in the HSQC, HMBC, and HR-ESI-MS spectra (Figures S21–S23), indicated that compound **3** was a demethylene derivative of 4-demethyl-dehydropodophyllotoxin [12]. The HMBC correlation (Figure 2 and Figure S22) between two methoxy groups at δ 3.72 (6H, s) and δ 147.5 (C-3, 5) indicated that they were located at C-3 and C-5. Thus, compound **3** was identified as 6′,7′-demethylene-4-demethyldehydropodophyllotoxin, and named dysoslignan C.

Compound **4** was obtained as a white amorphous powder and possessed a molecular formula C_20_H_14_O_8_, as revealed by its HR-ESI-MS analysis (*m*/*z* 383.0768 [M + H]^+^, calcd for 383.0767). The ^1^H NMR spectrum (Table 2 and Figure S24) showed one methoxy group at δ 3.70 (3H, s); four aromatic protons at δ 7.60 (1H, s), 6.89 (1H, s), 6.30 (1H, d, *J* = 1.9 Hz), and 6.28 (1H, d, *J* = 1.9 Hz); one methylenedioxy group at δ 6.151 (1H, s), 6.152 (1H, s); and one methylene group at δ 5.30 (2H, s). The ^13^C NMR spectrum (Table 2 and Figure S25) revealed a skeleton of arylnaphthalene lactone lignan including one carbonyl group at δ 169.5, sixteen aromatic carbons and one aliphatic carbon at δ 66.5, as well as one methoxyl group at δ 55.9, and one methylenedioxy group at δ 101.9. A careful comparison of the NMR spectra of **4** with 4-demethyl-dehydropodophyllotoxin, combined with data observed in the HSQC, HMBC, and HR-ESI-MS spectra (Figures S26–S28), suggested compound **4** to be a demethylation derivative of 4-demethyldehydropodophyllotoxin [12]. The HMBC correlation (Figure 2 and Figure S27) between the methoxy group at δ 3.70 (3H, s) and δ 148.0 (C-3), indicated that it was located at C-3. Thus, compound **4** was identified as 3,4-di-demethyldehydropodophyllotoxin, and named dysoslignan D.

All isolated compounds were evaluated for their in vitro cytotoxic activities against the A-549 and SMMC-7721 cell lines using the MTS assay [22] with cisplatin and paclitaxel as positive controls, and the IC_50_ values are summarized in Table 3. Compounds **7**–**12** and **14**–**17** showed more potent cytotoxicities against the SMMC-7721 cell line than the A549 cell line. Compounds **7**–**10** and **14**–**16** exhibited more potent activities than cisplatin in two tumor cell lines. Compound **14** showed the highest cytotoxicity against the A-549 and SMMC-7721 cell lines, with IC_50_ values of 0.130 and 0.0088 μM, respectively. The glycosylation of 7′-hydroxy group strongly reduced the activity; for example, comparing **16** to **15**, **17** to **14**, and **2**, **5**, and **6** to **7**. The cis-fusion compounds (**6**, **7** and **8**) between the tetraline and lactone were more cytotoxic than those corresponding trans-fusion analogues (**16**, **15**, and **14**). Compounds **7**, **15** and **8**, **14** containing a non-aromatized ring C exhibited more cytotoxic activity than aromatized compounds **9** and **10**, indicating that the non-aromatized ring C played an important role in the cytotoxicity against A-549 and SMMC-7721 cells lines. The methylenedioxy-bearing compound (**9**) was found to be more potent than the ring A-opened analogue (**3**). The preliminary structure-activity relationship investigation suggested that the trans-fusion between the tetraline and lactone, non-aromatized ring C, and a methylenedioxy at ring A, were structurally required for maintaining cytotoxicity for related podophyllotoxin analogues.

## 3. Experimental Section

### 3.1. General Experimental Procedures

Optical rotations and ECD spectra were determined by a Rudolph AP-IV polarimeter (Rudolph, Hackettstown, NJ, USA) and an Applied Photophysics Chirascanq CD spectropolarimeter (AppliedPhotophysics, Leatherhead, Surrey, UK), respectively. UV and IR spectra were obtained using a Thermo EVO 300 spectrometer (Thermo, Waltham, MA, USA) and a Thermo Nicolet IS 10 spectrometer (Thermo, Waltham, MA, USA), respectively. NMR and mass spectra were performed on a Bruker Avance III 500 spectrometer (Bruker, Rheinstetten, Germany) and a Bruker maXisHD mass spectrometer (Bruker, Bremen, Germany), respectively. Preparative HPLC separations were run on a SEP system (Beijing Sepuruisi scientific Co., Ltd., Beijing, China) equipped with a variable-wavelength UV detector, using a YMC-Pack ODS-A column (250 × 20 mm, 5 μm). ODS (50 μm), sephadex LH-20 (40–70 μm), and silica gel (160–200 mesh) were acquired from YMC Co. Ltd. (Kyoto, Japan), Amersham Pharmacia Biotech AB, (Uppsala, Sweden), and Marine Chemical Industry, (Qingdao, China), respectively. MCI gel CHP-20 and Diaion HP-20 were obtained from Mitsubishi Chemical Corp. (Tokyo, Japan). Chemical reagents for isolation were of analytical grade and purchased from Tianjin Siyou Co., Ltd., Tianjin, China. Biological reagents were from Sigma Company.

### 3.2. Plant Material

The roots and rhizomes of *D. versipellis* were collected in Qingzhen, Guizhou Province, China, in July 2019, and identified by Prof. Cheng-Ming Dong at School of Pharmacy, Henan University of Chinese Medicine, where a voucher specimen (DV 20190706) was deposited.

### 3.3. Extraction and Isolation

The powered roots and rhizomes of *D. versipellis* (40 kg) were refluxed with 95% EtOH (*v*/*v* 120 L × 3, 1.5 h each) and 50% EtOH (*v*/*v* 120 L × 1, 1.5 h each) at 95 ℃, respectively. The filtrate was evaporated under reduced pressure to give a dark brown residue (5.4 kg). The residue was adsorbed by silicious earth and eluted by CH_2_Cl_2_, EtOAc, and MeOH. The MeOH extract (3.4 kg) was fractioned by silica gel column chromatography (CC), eluting with a gradient of CH_2_Cl_2_–MeOH (*v*/*v* 100:0, 100:1, 100:3, 100:5, 100:7, 100:10, 100:30, 100:50, 0:100). Nine fractions M1~M9 were obtained on the basis of TLC monitoring results. The white precipitates (3.5 g) from fraction M4 was isolated by preparative HPLC (MeOH:H_2_O, 66:34) at a flow rate of 3 mL min^−1^ to give compounds **7** (t_R_ 13.7 min, 3.6 mg), **15** (t_R_ 16.0 min, 12.4 mg), **8** (t_R_ 18.3 min, 2.7 mg), **14** (t_R_ 21.2 min, 2.7 mg), **9** (t_R_ 28.1 min, 5.2 mg), and **10** (t_R_ 44.5 min, 3.0 mg). Fraction M4 (90.2 g) was subjected to sephadex LH-20 CC eluted by methanol to yield subfractions M4–1~M4–3. Subfraction M4–1 (23.7 g) was submitted to ODS CC eluted by MeOH–H_2_O (10:90, 30:70, 50:50, 70:30, 90:10, 100:0) to afford subfractions M4–1–1~M4–1–6. Subfraction M4–1–2 (4.8 g) was separated by sephadex LH-20 CC eluted by methanol to yield subfractions M4–1–2–1~M4–1–2–6. Subfraction M4–1–2–3 (1.6 g) was isolated by preparative HPLC (MeOH:H_2_O, 52:48) at a flow rate of 3 mL min^−1^ to give subfractions M4–1–2–3–1 (t_R_ 7.9 min), M4–1–2–3–2 (t_R_ 8.3 min), M4–1–2–3–3 (t_R_ 10.8 min), M4–1–2–3–4 (t_R_ 12.8 min), M4–1–2–3–5 (t_R_ 16.4 min), and M4–1–2–3–6 (t_R_ 25.1 min). Subfraction M4–1–2–3–2 (22.7 mg) was purified by preparative HPLC (MeOH:H_2_O, 48:52) at a flow rate of 3 mL min^−1^ to afford **3** (tR 11.9 min, 2.5 mg). Subfraction M4–1–2–3–5 (19.2 mg) was isolated by preparative HPLC (MeOH:H_2_O, 42:58) at a flow rate of 3 mL min^−1^ to afford **11** (t_R_ 54.1 min, 2.1 mg). Subfraction M4–1–2–3–6 (25.3 mg) was purified by preparative HPLC (MeOH:H_2_O, 50:50) at a flow rate of 3 mL min^−1^ to afford **4** (t_R_ 32.5 min, 2.2 mg). Fraction M5 (110.0 g) was subjected to sephadex LH-20 CC eluted by methanol to yield subfractions M5–1~M5–8. Subfractions M5–1~M5–5 (22.9 g) were combined and submitted to MCI CC eluted by MeOH–H_2_O (0:100, 10:90, 30:70, 50:50, 70:30, 90:10, 100:0) to afford subfractions M5–1–1~M5–1–4. Subfraction M5–1–2 (3.7 g) was applied to silica gel CC with a CHCl_3_-MeOH (100:0, 100:1, 100:3, 100:5 100:7, 100:10, 7:1, 3:1) gradient to give subfractions M5–1–2–1~M5–1–2–8. Subfraction M5–1–2–6 (50 mg) was isolated by preparative HPLC (MeOH:H_2_O, 54:46) at a flow rate of 3 mL min^–1^ to give compound **16** (t_R_ 18.5 min, 5.6 mg). Subfraction M5–1–3 (0.25 g) was separated by silica gel CC with a CHCl_3_-MeOH (100:0, 100:1, 100:3, 100:5 100:7, 100:10, 7:1, 3:1) gradient to give subfractions M5–1–3–1~M5–1–3–9. Subfraction M5–1–3–5 (2.5 g) was purified by preparative HPLC (MeOH:H_2_O, 55:45) at a flow rate of 3 mL min^–1^ to give compounds **12** (t_R_ 49.0 min, 4.3 mg) and **17** (t_R_ 32.2 min, 3.3 mg). The precipitates from subfraction M5 were washed repeatedly by MeOH, and then the white powder (compound **6**) was obtained. Fraction M6 (130.0 g) was subjected to sephadex LH-20 CC eluted by methanol to yield subfractions M6–1 and M6–2. The subfraction M6–1 (57.3 g) was applied to ODS CC with a MeOH-H_2_O (10:90, 30:70, 50:50, 70:30, 90:10, 100:0) gradient to give subfractions M6–1–1~M6–1–4. Subfraction M6–1–2 (5.8 g) was separated by silica gel CC with a CH_2_Cl_2_-MeOH (100:0, 100:1, 100:3, 100:5, 100:7, 100:10, 100:30) gradient to give subfractions M6–1–2–1~M6–1–2–7. Subfraction M6–1–2–4 (1.03 g) was isolated by preparative HPLC (MeOH:H_2_O, 45:55) at a flow rate of 3 mL min^–1^ to give subfraction M6–1–2–4–1 (t_R_ 11.0 min) and **2** (t_R_ 39.1 min, 6.6 mg). Subfraction M6–1–2–4–1 (15.2 mg) was applied to preparative HPLC (MeOH:H_2_O, 38:62) at a flow rate of 3 mL min^−1^ to give **18** (t_R_ 17.0 min, 3.2 mg). The subfraction M6–1 (57.3 g) was applied to ODS CC with a MeOH-H_2_O (10:90, 30:70, 50:50, 70:30, 90:10, 100:0) gradient to give subfractions M6–1–1~M6–1–4. Subfraction M6–2 (65.9 g) was separated by silica gel CC with a CH_2_Cl_2_-MeOH (100:1, 100:3, 100:5, 100:7, 100:10, 100:30) gradient to give subfractions M6–2–1~M6–2–6. Subfraction M6–2–5 (1.7 g) was submitted to preparative HPLC (MeOH:H_2_O, 60:40) at a flow rate of 3 mL min^−1^ to give **13** (t_R_ 22.9 min, 4.0 mg). The subfractions M7 and M8 were combined and then applied to Diaion HP-20 CC with an EtOH-H_2_O (10:90, 30:70, 50:50, 70:30, 90:10, 100:0) gradient to give subfractions M7–1~M7–6. The white sticky gum from subfraction M7–2 was washed repeatedly by MeOH and then separated by preparative HPLC (MeOH:H_2_O, 35:65) at a flow rate of 3 mL min^−1^ to give compounds **1** (t_R_ 16.5 min, 10.0 mg) and **5** (t_R_ 21.7 min, 3.7 mg).

### 3.4. Spectroscopic and Physical Data

Dysoslignan A (**1**): white, amorphous powder; [α${]}_{D}^{20}$–24.6 (c 0.28, MeOH); ECD (MeOH) λmax (Δε) 206 (–15.0), 222 (+0.5), 237 (–2.0), 285 (–0.3) nm; UV (MeOH) λmax (log ε) 204 (4.81), 275 (3.79), 285 (3.66) nm; IR (iTR)*ν*_max_ 3386, 2931, 2905, 2832, 1759, 1653, 1594, 1506, 1462, 1447, 1422, 1389, 1334, 1244, 1192, 1169, 1127, 1074, 1037 cm^−1^; HR-ESI-MS (positive): *m*/*z* 763.2419 [M + Na]^+^ (calcd for C_34_H_44_O_18_Na, 763.2425); NMR data (DMSO-*d*_6_), see Table 1.

Dysoslignan B (**2**): white, amorphous powder; [α${]}_{D}^{20}$–39.7 (c 0.25, MeOH); ECD (MeOH) λmax (Δε) 208 (+3.75), 238 (+0.31), 290 (+0.42) nm; UV (MeOH) λmax (log ε) 204 (4.45), 242 (3.62), 284 (3.36) nm; IR (iTR)*ν*_max_ 3381, 2361, 1764, 1616, 1523, 1475, 1375, 1335, 1264, 1219, 1168, 1121, 1033 cm^−1^; HR-ESI-MS (positive): *m*/*z* 909.2637 [M + Na]^+^ (calcd for C_39_H_50_O_23_Na, 909.2641); NMR data (DMSO-*d*_6_), see Table 1.

Dysoslignan C (**3**): white, amorphous powder; UV (MeOH) λmax (log ε) 204 (4.49), 225 (4.19), 264 (4.35), 326 (3.76), 363 (3.60) nm; IR (iTR)*ν*_max_ 3367, 2989, 2946, 2833, 1741, 1608, 1520, 1467, 1420, 1348, 1274, 1213, 1186, 1115, 1090, 1027 cm^−1^; HR-ESI-MS (positive): *m*/*z* 385.0920 [M + H]^+^ (calcd for C_20_H_17_O_8_, 385.0923), *m*/*z* 407.0737 [M + Na]^+^ (calcd for C_20_H_16_O_8_Na, 407.0743); NMR data (DMSO-*d*_6_), see Table 2.

Dysoslignan D (**4**): white, amorphous powder; UV (MeOH) λmax (log ε) 202 (4.48), 225 (4.29), 263 (4.38), 312 (3.82), 355 (3.60) nm; IR (iTR)*ν*_max_ 3410, 2939, 2839, 1745, 1605, 1535, 1465, 1352, 1244, 1131, 1094, 1030 cm^−1^; HR-ESI-MS (positive): *m/z* 383.0768 [M + H]^+^ (calcd for C_20_H_15_O_8_, 383.0767); NMR data (DMSO-*d*_6_), see Table 2.

### 3.5. Acid Hydrolysis and Sugar Determination

The absolute configurations of the galatose and glucose moieties were determined by the previously reported method [19]. Compounds **1** (1.0 mg) and **2** (1.0 mg) were dissolved in 1.0 mL of 2M HCl, and then hydrolyzed at 90 °C for 3 h. The HCl in the reaction mixture was removed under reduced pressure. The remaining reaction mixture was extracted with CH_2_Cl_2_. The water layers were directly analyzed by HPLC [column: Asahipak NH_2_P-50 4E (4.6 mm × 250 mm); mobile phase: CH_3_CN-H_2_O (17:3), flow rate: 0.7 mL/min]. The peaks at 13.15 and 14.27 min were coincided with D-glucose and D-galatose.

### 3.6. Cytotoxicity Asssay

By the previously reported MTS method [22], the cytotoxic activities of compounds **1**–**18** were evaluated against human lung cancer A-549, hepatocellular carcinoma SMMC-7721 cell lines. The cells were cultured in RPMI-1640 medium, supplemented with 10% fetal bovine serum (FBS) at 37 ℃ under 5% CO_2_ in a humidified atmosphere. Cell viability was assessed by conducting colorimetric measurements of the amount of insoluble formazan formed in living cells based on the reduction of 3-(4,5-dimethylthiazol-2-yl)-5-(3-carboxymethoxyphenyl)-2-(4-sulfophenyl)-2H-tetrazolium (MTS). To be brief, 100 µL of cells were seeded into each well in a 96-well cell culture plate in advance. After 24 h, various concentrations of all test compounds were added. After the incubation for 48 h, MTS (20 μL) was added to each well, and the incubation continued for 4 h at 37 ℃. The optical density at 492 nm was determined using a 96-well microtiter plate reader. The IC_50_ values were calculated by the Reed–Muench method. Statistical analysis were performed by SPSS 20.0 software (SPSS Inc., Chicago, IL, USA). All experiments were performed in triplicate.

## 4. Conclusions

Further phytochemical studies on *D. versipellis* resulted in the isolation of one new dibenzyltyrolactone lignan dysoslignan A (**1**), three new arylnaphthalide lignans dysoslignan B–C (**2**–**4**), along with fourteen known metabolites (**5**–**18**). Compound **2** is the first reported example of naturally occurring arylnaphthalide lignan triglycoside. All isolated compounds were tested for their in vitro cytotoxic activity against A-549 and SMMC-7721 cell lines using MTS assay. Among them, compounds **7**, **14**, and **15** were cytotoxic, with IC_50_ values of less than 1μM. Our research further demonstrated that the arylnaphthalide lignans are mainly responsible for the potent anticancer effect of *D. versipellis.*

## Figures and Tables

**Table 1:** ^1^H NMR (500 MHz) and ^13^ C NMR (125 MHz) data (DMSO-*d*_6_) of **1**–**2.**

NO.	1		2		NO.	1		2	
	δ_H_	δ_C_	δ_H_	δ_C_		δ_H_	δ_C_	δ_H_	δ_C_
1		134.1 C		132.2 C	4-OCH_3_	3.63, s	59.9		
2	6.76, s	103.6 CH	6.57, s	106.6 CH	1″	4.14, d (7.8)	99.8 CH	4.51, d (7.4)	103.6 CH
3		152.9 C		148.1 C	2″	3.13, m	73.5 CH	3.19, m	73.9 CH
4		136.3 C		134.2 C	3″	3.20, m	76.5 CH	3.24, m	77.0 CH
5		152.9 C		148.1 C	4″	3.20, m	81.0 CH	3.07, m	70.4 CH
6	6.76, s	103.6 CH	6.57, s	106.6 CH	5″	3.20, m	76.3 CH	3.42, m	76.9 CH
7	5.23, d (2.7)	76.8 CH	3.83, d (8.1)	43.3 CH	6″	3.56, m; 3.68, m	61.0 CH_2_	3.89, m; 3.41, m	68.4 CH_2_
8	2.64, dd (2.7, 5.3)	51.7 CH	3.43, overlapped	43.4 CH	1‴	4.26, d (7.9)	103.2 CH	4.12, d (8.3)	103.4 CH
9		176.7 C		178.1 C	2‴	2.97, m	73.3 CH	2.96, m	73.6 CH
1′		132.2 C		132.3 C	3‴	3.17, m	74.6 CH	3.12, m	76.8 CH
2′	6.40, d (1.0)	108.7 CH	7.23, s	129.9 CH	4‴	3.06, m	70.8 CH	3.04, m	70.1 CH
3′		147.1 C		145.5 C	5‴	3.17, m	74.7 CH	3.18, m	76.8 CH
4′		145.5 C		146.2 C	6‴	3.73, m; 3.39, m	61.1 CH_2_	3.90, m; 3.55, m	68.3 CH_2_
5′	6.64, d (7.9)	107.8 CH	5.95, s	107.5 CH	1⁗			4.10, d (8.0)	103.2 CH
6′	6.35, dd (7.9, 1.0)	121.5 CH		132.8 C	2⁗			2.85, m	73.5 CH
7′	2.46, dd (13.6, 8.0) 2.09, dd (13.6, 7.5)	38.6 CH_2_	4.61, d (10.0)	76.6 CH	3⁗			2.56, m	76.4 CH
8′	2.85, m	36.0 CH	2.77, m	42.1 CH	4⁗			3.01, m	69.8 CH
9′	3.95, dd (8.6, 4.9) 4.30, t (8.2)	72.1 CH_2_	4.57, d (9.3); 4.42, dd (8.5, 7.0)	69.0 CH_2_	5⁗			2.83, m	75.0 CH
OCH_2_O	5.92, s; 5.94, s	100.7	5.94, s; 5.86, s	100.9	6⁗			3.65, m;3.43, m	61.1 CH_2_
3,5-OCH_3_	3.73, s	55.5	3.73, s	56.2					

**Table 2:** ^1^H NMR (500 MHz) and ^13^C NMR (125 MHz) data (DMSO-*d*_6_) of **3**–**4.**

NO.	3		4		NO.	3		4	
	δ_H_	δ_C_	δ_H_	δ_C_		δ_H_	δ_C_	δ_H_	δ_C_
1		125.8 C		125.3 C	3′		148.5 C		148.7 C
2	6.48, s	108.1 CH	6.30, d (1.9)	105.9 CH	4′		147.5 C		148.2 C
3		147.5 C		148.0 C	5′	7.03, s	109.6 CH	6.89, s	102.8 CH
4		134.9 C		133.6 C	6′		129.7 C		131.2 C
5		147.5 C		145.2 C	7′		144.1 C		145.2 C
6	6.48, s	108.1 CH	6.28, d (1.9)	111.2 CH	8′		120.0 C		119.0 C
7		130.3 C		130.2 C	9′	5.30, s	66.5 C	5.30, s	66.5 C
8		117.3 C		122.4 C	OCH_2_O			6.151, s; 6.152, s	101.9
9		169.9 C		169.5 C	3-OCH_3_	3.72, s	56.0	3.70, s	55.9
1′		123.5 C		124.7 C	5-OCH_3_	3.72, s	56.0		
2′	7.54, s	104.3 CH	7.60, s	98.0 CH					

**Table 3:** Cytotoxicities of compounds **1**–**18** against A549 and SMMC-7721 cell lines (IC_50_, μM) ^a^.

No.	A549	SMMC-7721	NO.	A549	SMMC-7721
**7**	0.306 ± 0.011	0.209 ± 0.014	**15**	0.182 ± 0.008	0.040 ± 0.002
**8**	7.72 ± 0.38	4.582 ± 0.37	**16**	8.48 ± 0.085	4.99 ± 0.26
**9**	15.55 ± 0.91	6.53 ± 0.48	**17**	30.92 ± 1.47 ^b^	6.07 ± 0.34
**10**	13.58 ± 0.06	5.424 ± 0.413	**1–6, 13, 18**	>40	>40
**11**	35.05 ± 0.28	23.71 ± 0.90	**cisplatin**	29.78 ± 0.82	12.23 ± 0.79
**12**	>40	36.20 ± 1.78	**paclitaxel**	<0.008	0.576 ± 0.057
**14**	0.130 ± 0.007	0.0088 ± 0.0002			

^a^ IC_50_ is expressed as mean ± SD of at least three determinations. ^b^ Significant differences are indicated as: *p* < 0.05 compared with cisplatin.

## Data Availability

Data are contained within the manuscript.

## Supplementary Materials

The following supporting information can be downloaded at: https://www.mdpi.com/article/10.3390/molecules28072909/s1: Figures S1–S28: NMR and HR-ESI-MS spectra of compounds **1**–**4,** and ECD spectra of compounds **1**–**2**.

## Sample Availability

Isolated compounds are not available from the authors.
