# Supplementary material for: Lignans from the Roots and Rhizomes of Dysosma versipellis and Their Cytotoxic Activities
Source: Molecules. 2023 Mar 24;28(7):2909. doi: 10.3390/molecules28072909 (PMC10095701; doi:10.3390/molecules28072909)
Supplement: Supplementary file 1 [file molecules-28-02909-s001.zip › molecules-2242566-supplementary.pdf]

# Lignans from the roots and rhizomes of *Dysosma versipellis* and their cytotoxic activities

**Yanjun Sun [1,2,3*], Haojie Wang [1,2], Ruijie Han [1,2], Hongyun Bai [1,2], Meng Li [1,2], Junmin Wang [1,2], and Weisheng Feng [1,2*]**

1 Collaborative Innovation Center for Respiratory Disease Diagnosis and Treatment & Chinese Medicine, Development of Henan Province, Henan University of Chinese Medicine, Zhengzhou 450046, China
2 School of Pharmacy, Henan University of Chinese Medicine, Zhengzhou 450046, China
3 Henan Research Center for Special Processing Technology of Chinese Medicine, 450046, China

* Correspondence: sunyanjun2011@hactcm.edu.cn (Y.S.); fwsh@hactcm.edu.cn (W.F.);
Tel.: +86-371-6596-2746 (Y.S. & W.F.)

**Abstract**: One new dibenzyltyrolactone lignan dysoslignan A (**1**), three new arylnaphthalide lignans dysoslignan B−C (**2**−**4**), along with fourteen known metabolites (**5**−**18**), were isolated from the roots and rhizomes of *Dysosma versipellis*. Their structures and stereochemistry were determined from analysis of NMR spectroscopic and circular dichroism (CD) data. Compound **2** represents the first report of naturally occurring arylnaphthalide lignan triglycoside. The cytotoxic activities of all isolated compounds were evaluated against A-549 and SMMC-7721 cell lines. Compounds **7**−**10**, and **14**−**16** were more toxic than cisplatin in two tumor cell lines. This investigation clarifies the potential effective substance basis of *D. versipellis* in tumor treatment.

**Keywords:** *Dysosma versipellis*; dibenzyltyrolactone; arylnaphthalide; cytotoxic

## Table of Contents

Figure S1. $^1$H NMR spectrum (500 MHz) of **1** in DMSO-*d6*........3
Figure S2. $^{13}$C NMR spectrum (125 MHz) of **1** in DMSO-*d6*........3
Figure S3. HSQC spectrum of **1**........4
Figure S4. HMBC spectrum of **1**........4
Figure S5 DEPT spectrum of **1**........5
Figure S6 $^1$H-$^1$H-COSY spectrum of **1**........5
Figure S7. NOESY spectrum of **1**........6
Figure S8. ECD spectrum of **1**........6
Figure S9. HR-ESI-MS spectrum of **1**........7
Figure S10. $^1$H NMR spectrum (500 MHz) of **2** in DMSO-*d6*........7
Figure S11. $^{13}$C NMR spectrum (125 MHz) of **2** in DMSO-*d6*........8
Figure S12. HSQC spectrum of **2**........8
Figure S13. HMBC spectrum of **2**........9
Figure S14. DEPT spectrum of **2**........9
Figure S15. $^1$H-$^1$H-COSY spectrum of **2**........10
Figure S16. NOESY spectrum of **2**........10
Figure S17. ECD spectrum of **2**........11
Figure S18. HR-ESI-MS spectrum of **2**........11
Figure S19. $^1$H NMR spectrum (500 MHz) of **3** in DMSO-*d6*........12
Figure S20. $^{13}$C NMR spectrum (125 MHz) of **3** in DMSO-*d6*........12
Figure S21. HSQC spectrum of **3**........13
Figure S22. HMBC spectrum of **3**........13
Figure S23. HR-ESI-MS spectrum of **3**........14
Figure S24. $^1$H NMR spectrum (500 MHz) of **3** in DMSO-*d6*........14
Figure S25. $^{13}$C NMR spectrum (125 MHz) of **3** in DMSO-*d6*........15
Figure S26. HSQC spectrum of **4**........15
Figure S27. HMBC spectrum of **4**........16
Figure S28. HR-ESI-MS spectrum of **4**........16

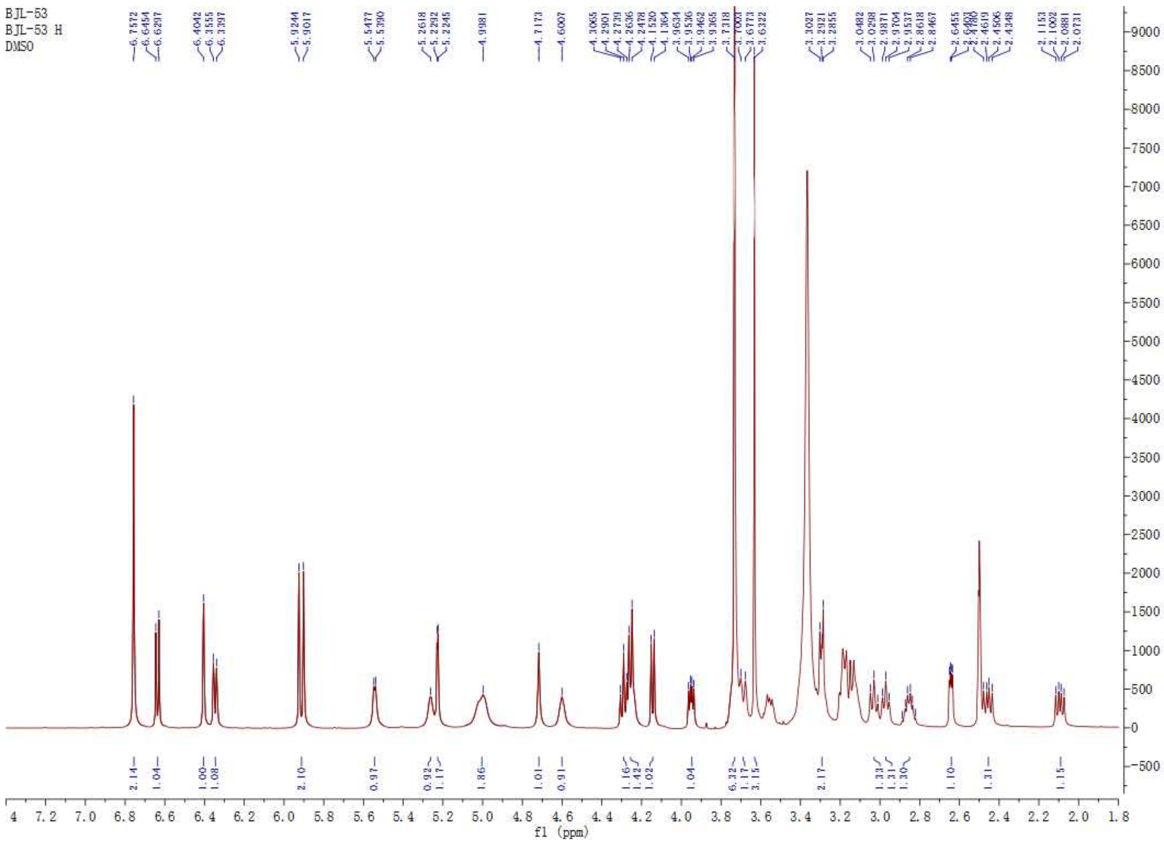

Figure S1. $^1$H NMR spectrum of **1** (500 MHz, DMSO-*d*6)

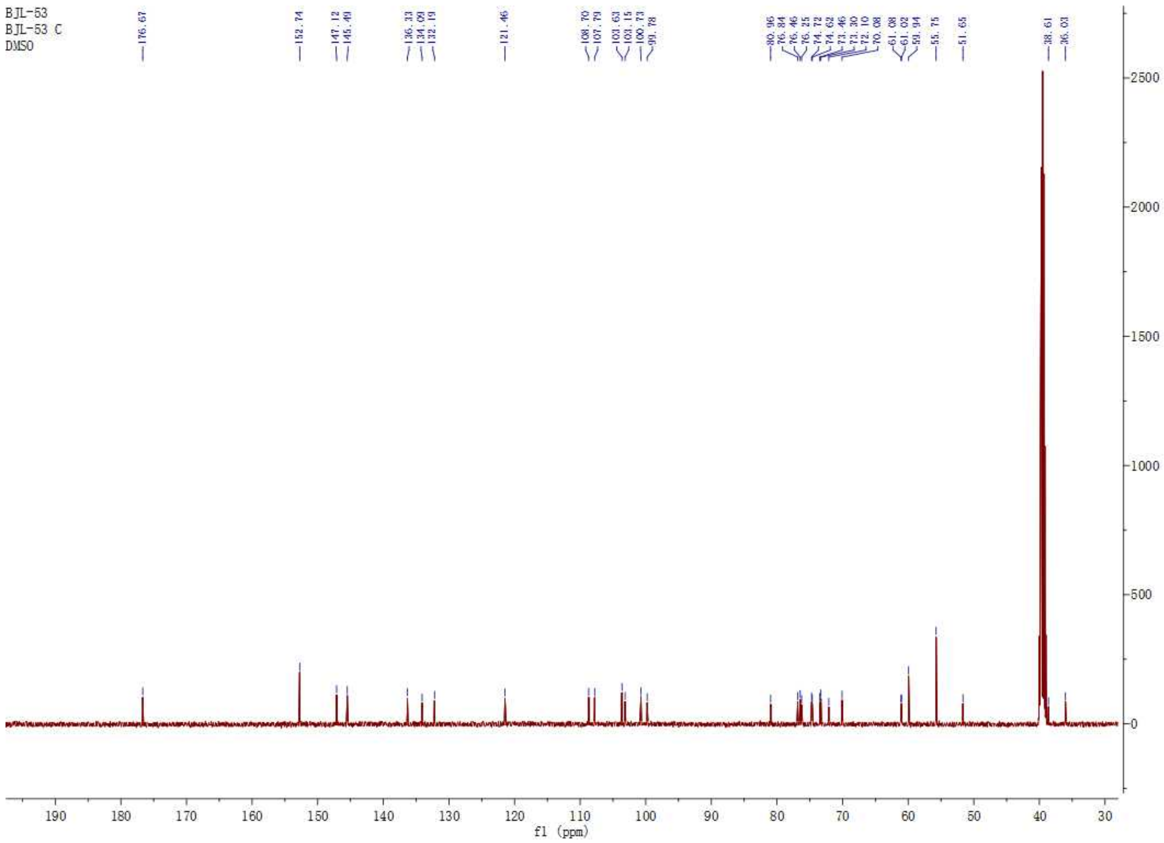


Figure S2. $^{13}$C NMR spectrum of **1** (125 MHz, DMSO-*d*6)

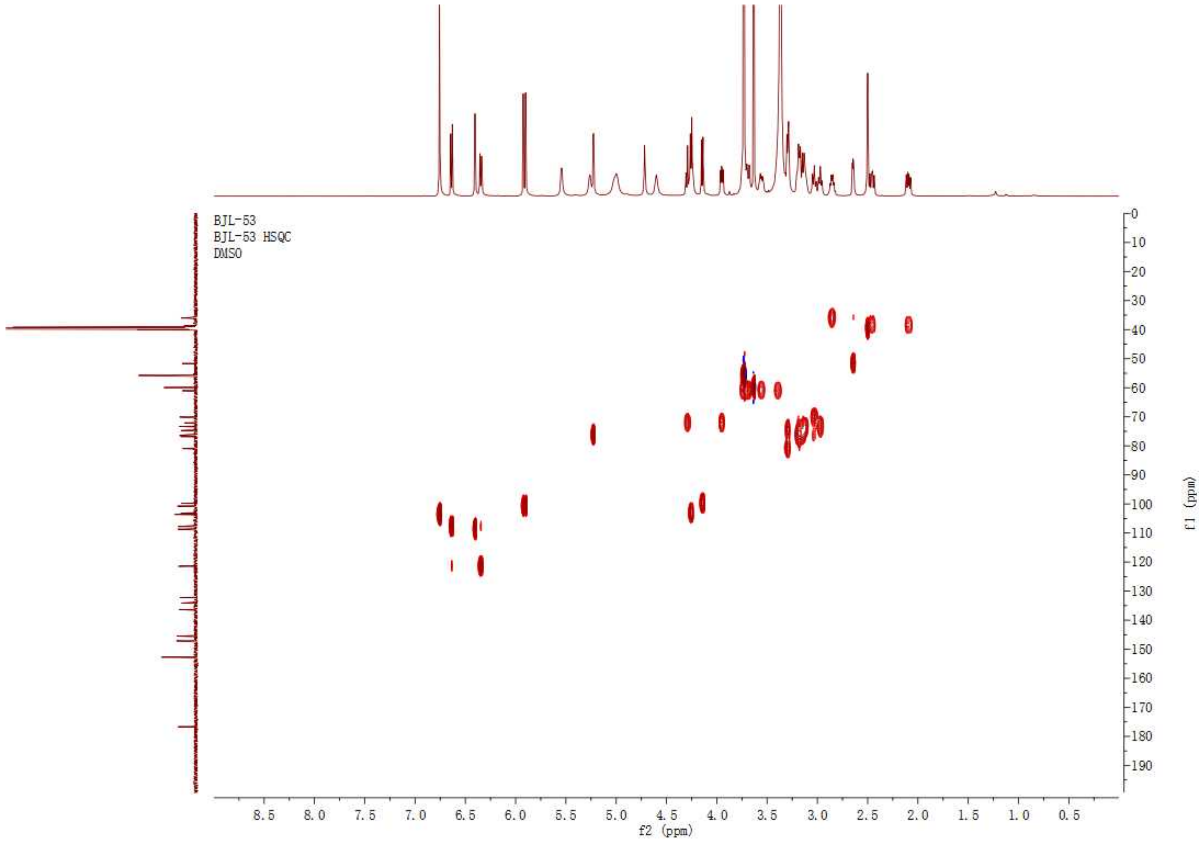


Figure S3. HSQC spectrum of **1**

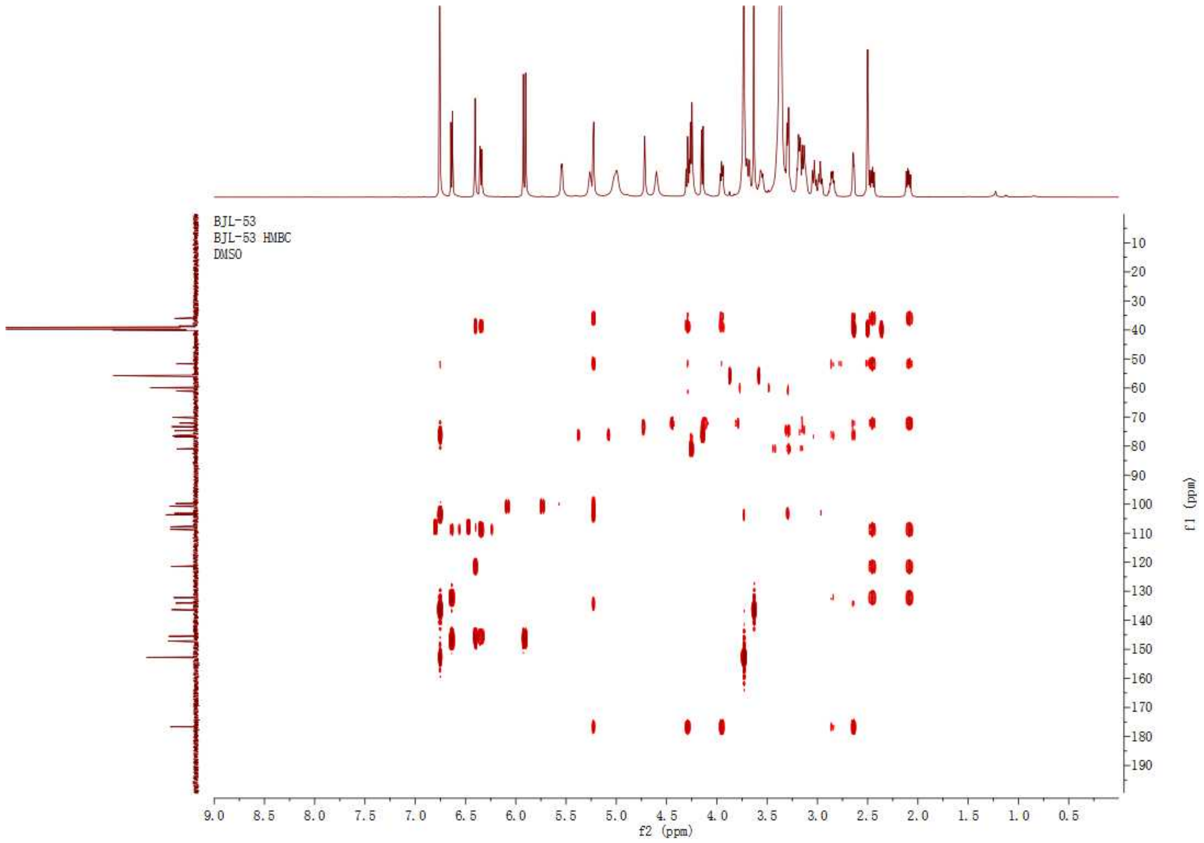


Figure S4. HMBC spectrum of **1**

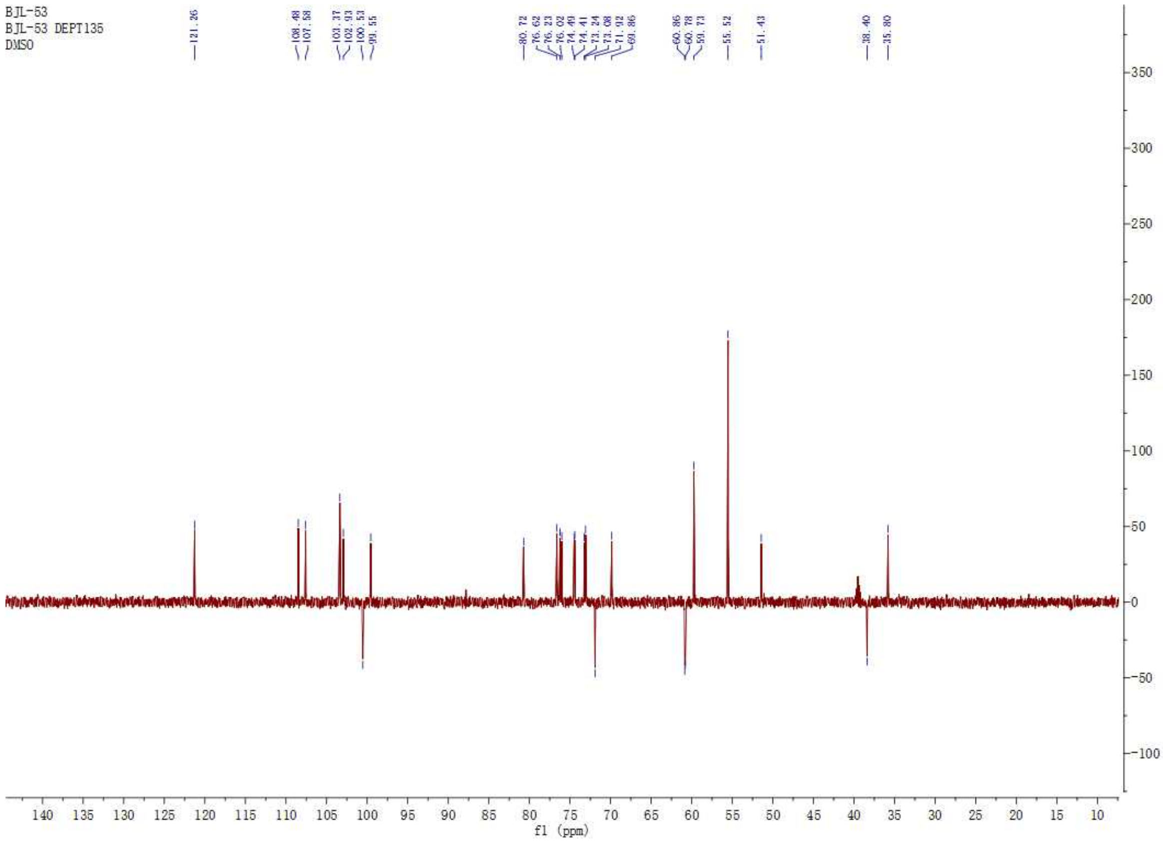


Figure S5. DEPT spectrum of **1**

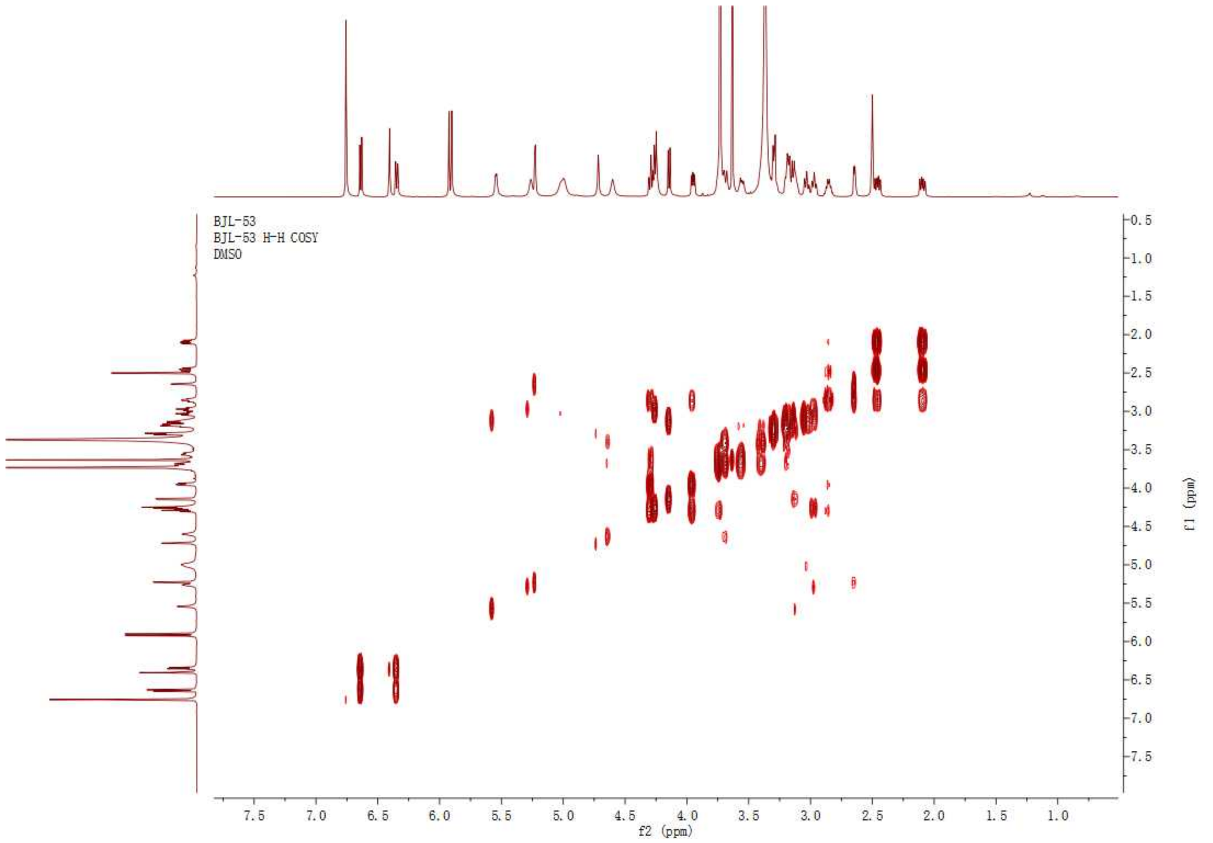


Figure S6. $^{1}H$-$^{1}H$-COSY spectrum of **1**

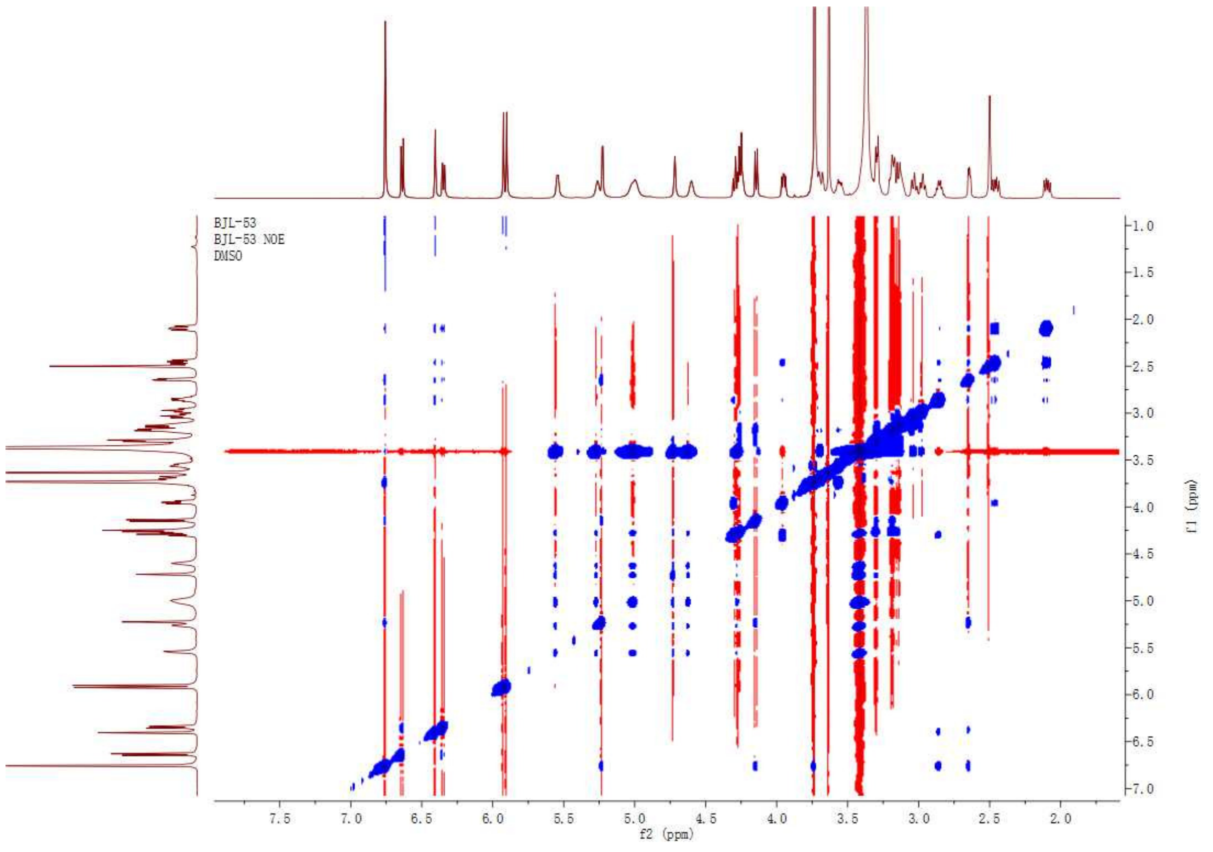

Figure S7. NOESY spectrum of **1**

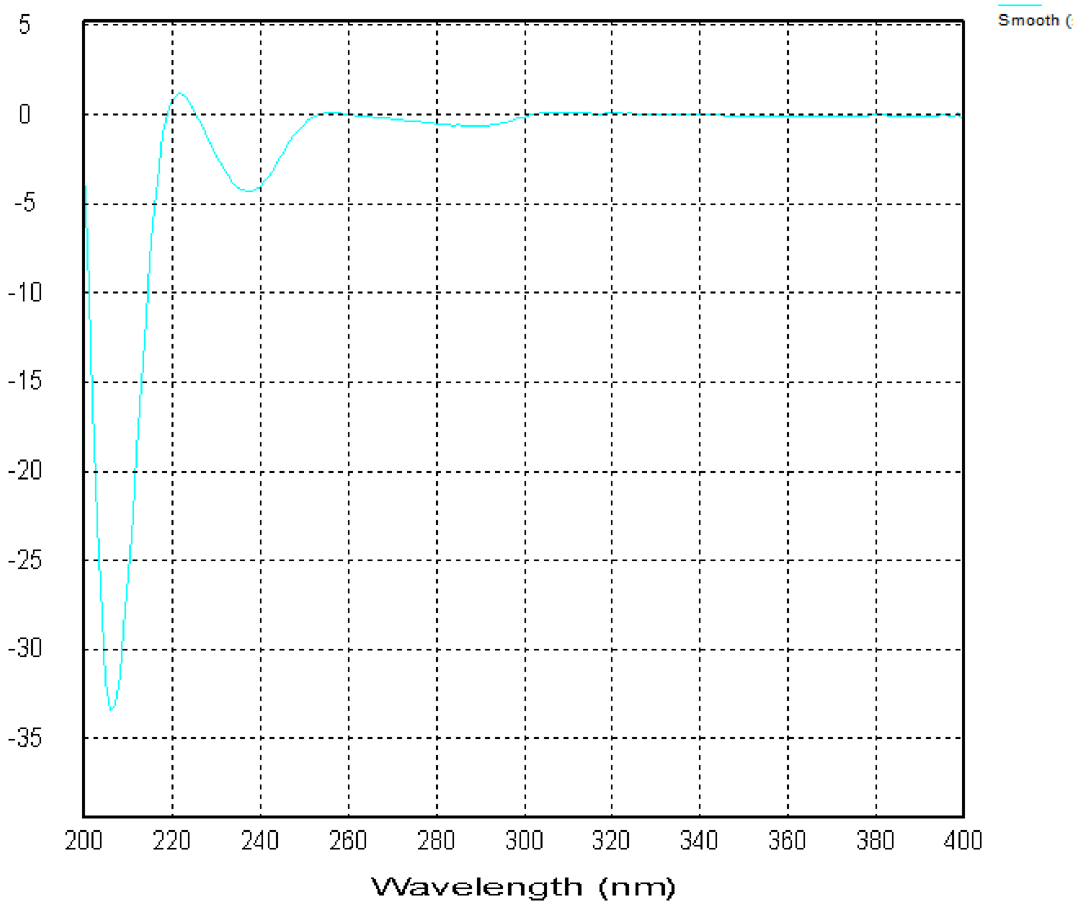


Figure S8. ECD spectrum of **1**

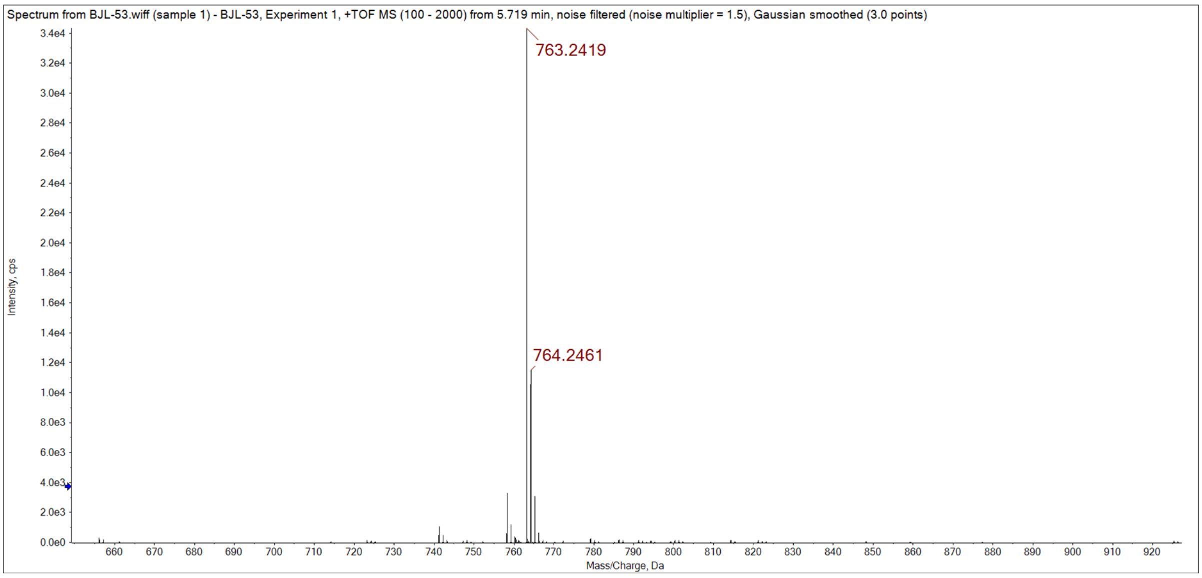


Figure S9. HR-ESI-MS spectrum of **1**

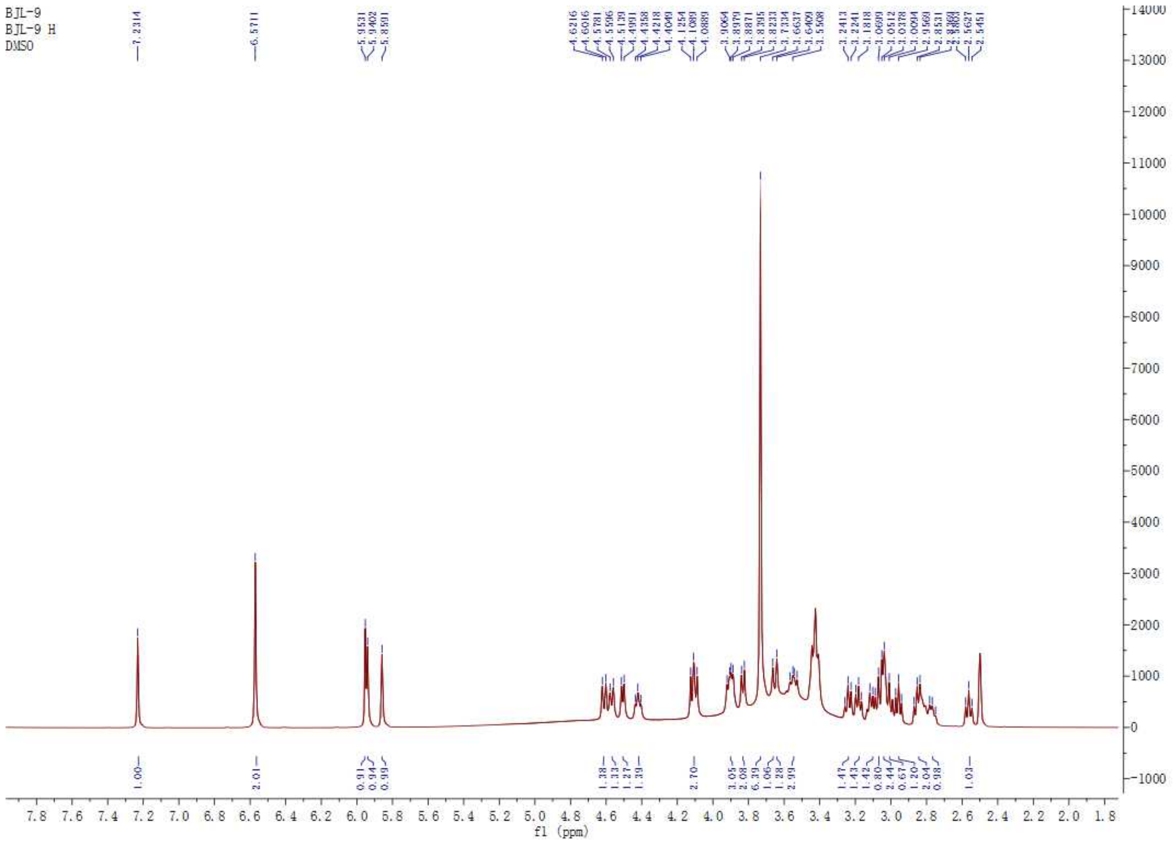

Figure S10. $^{1}$H NMR spectrum of **2** (500 MHz, DMSO-*d6*)

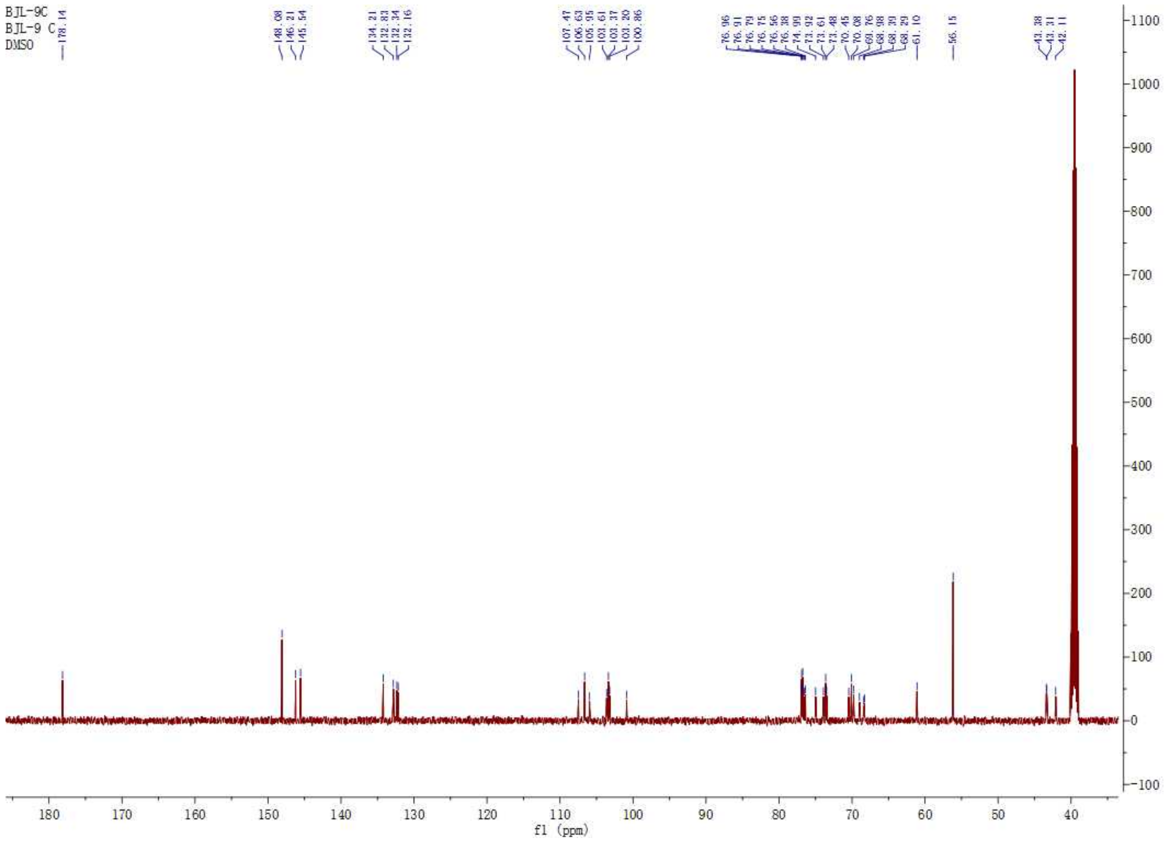

Figure S11. $^{13}$C NMR spectrum of **2** (125 MHz, DMSO-*d*6)

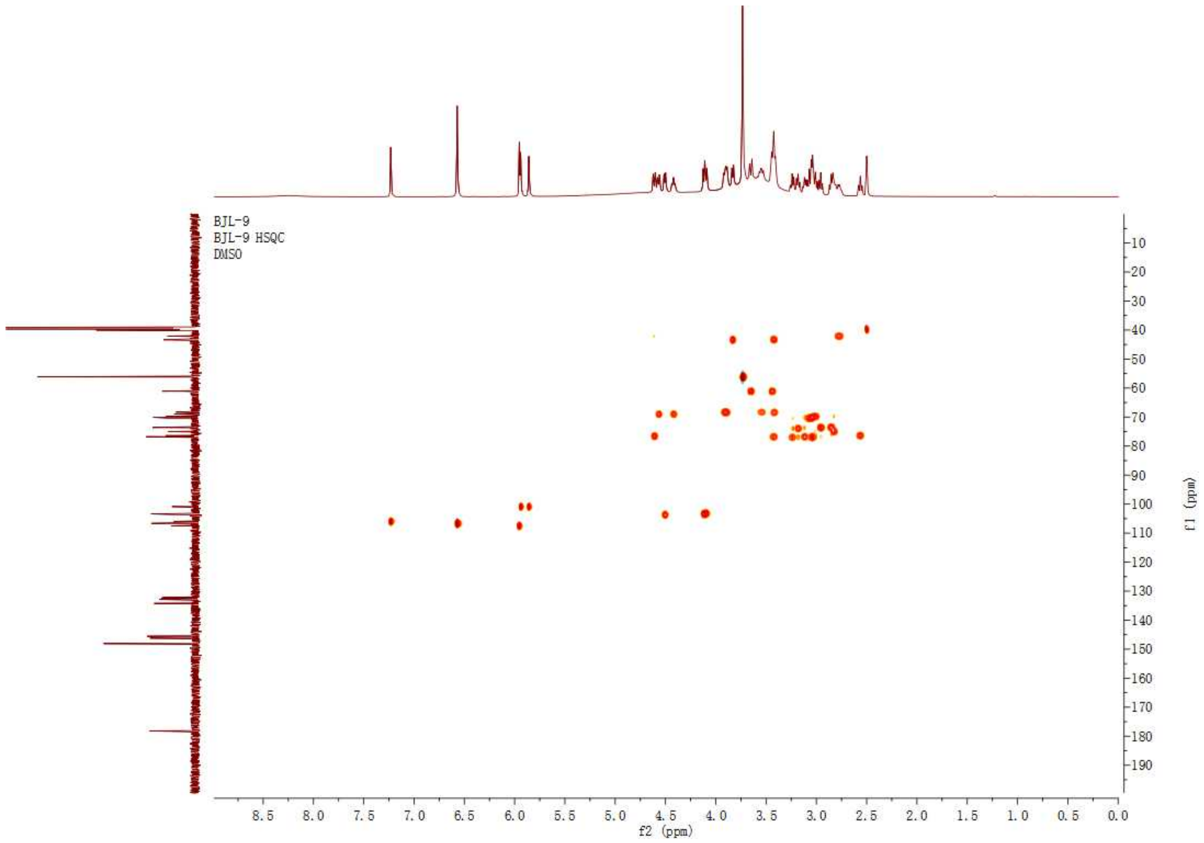


Figure S12. HSQC spectrum of **2**

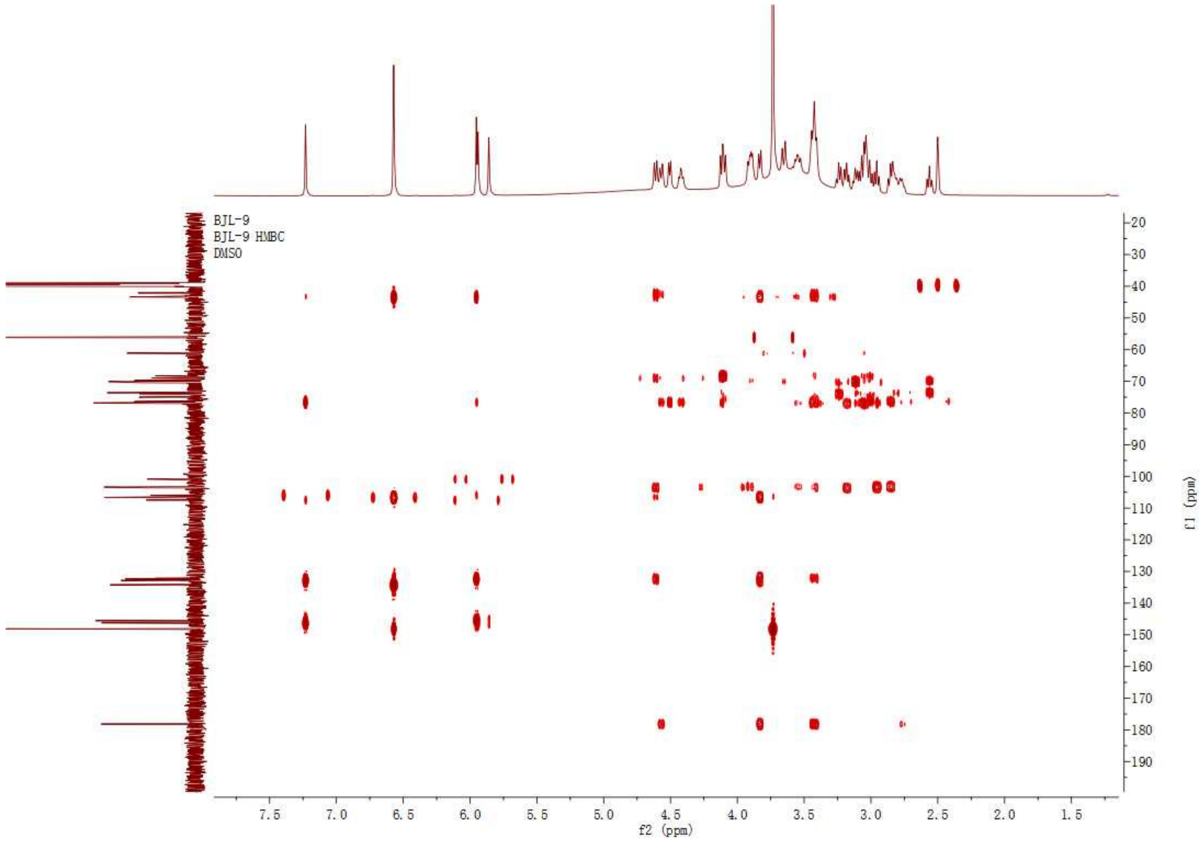

Figure S13. HMBC spectrum of **2**

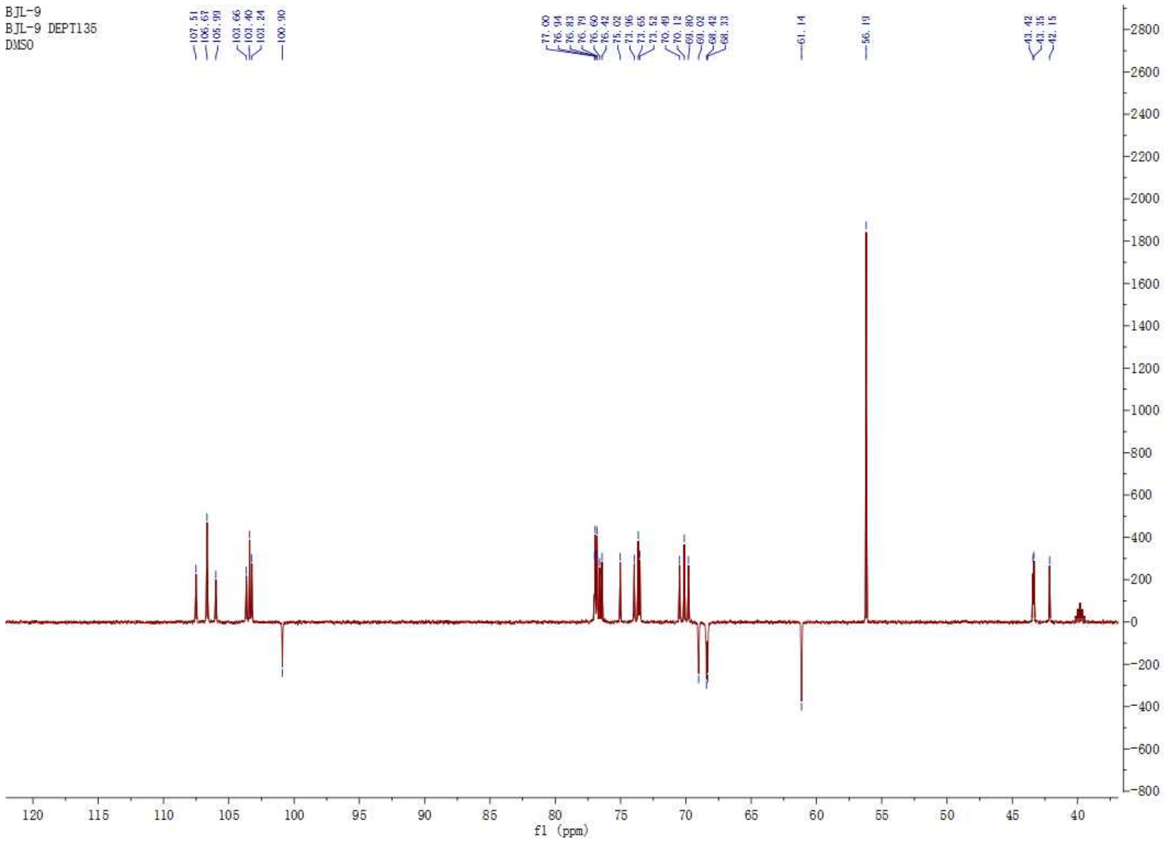


Figure S14. DEPT spectrum of **2**

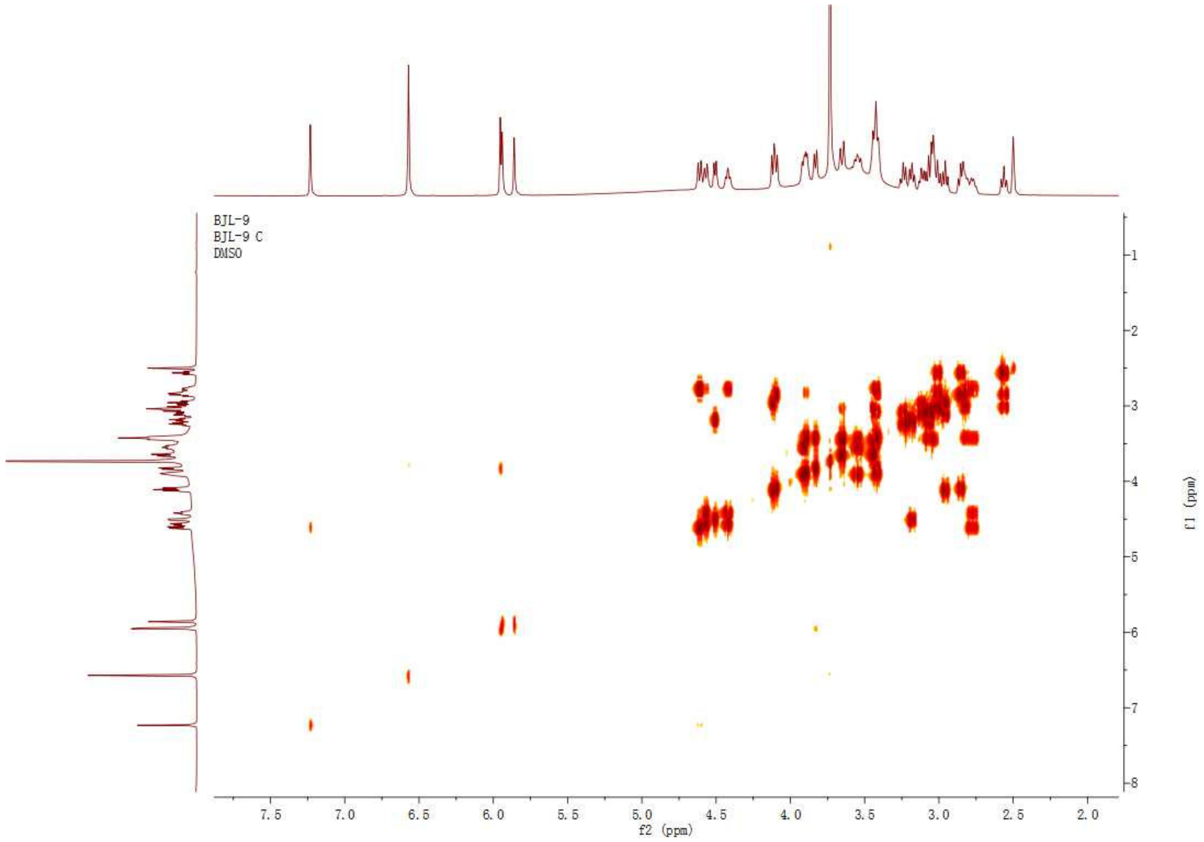

Figure S15. $^{1}$H-$^{1}$H-COSY spectrum of **2**

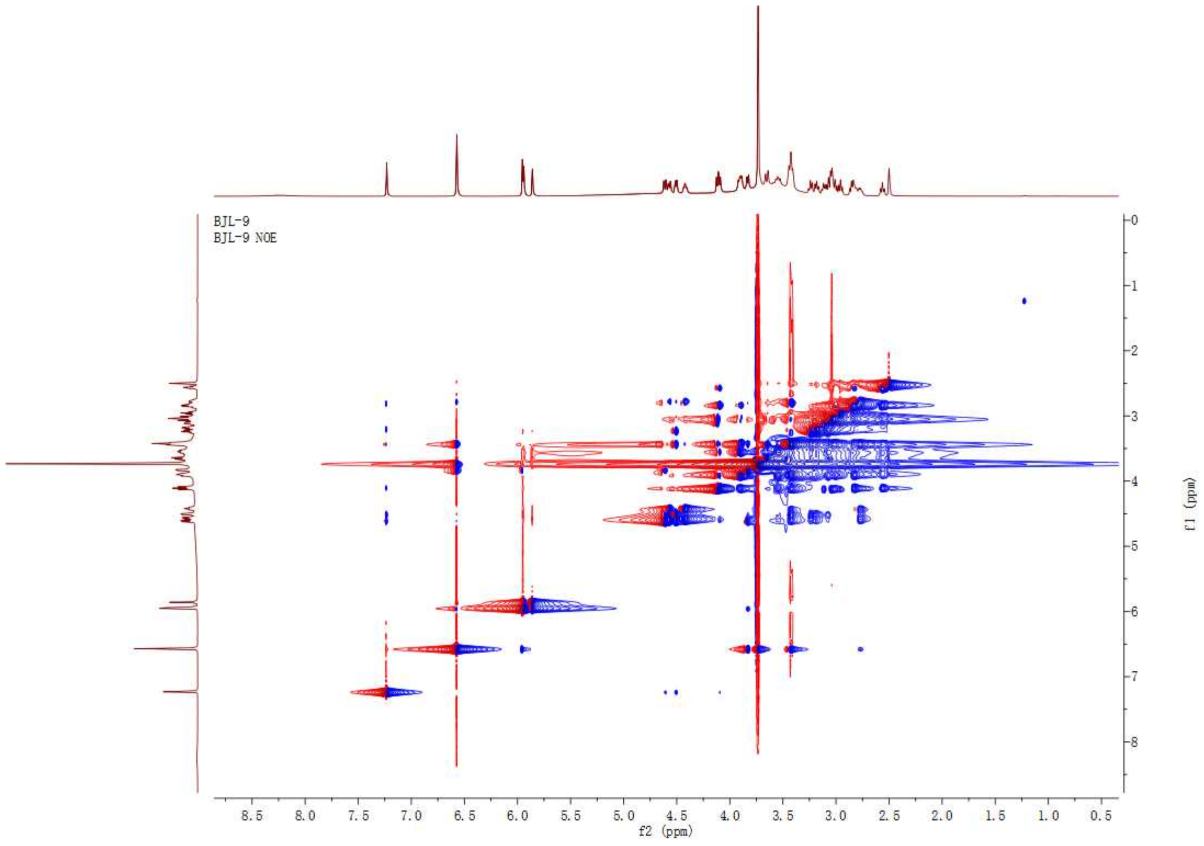


Figure S16. NOESY spectrum of **2**

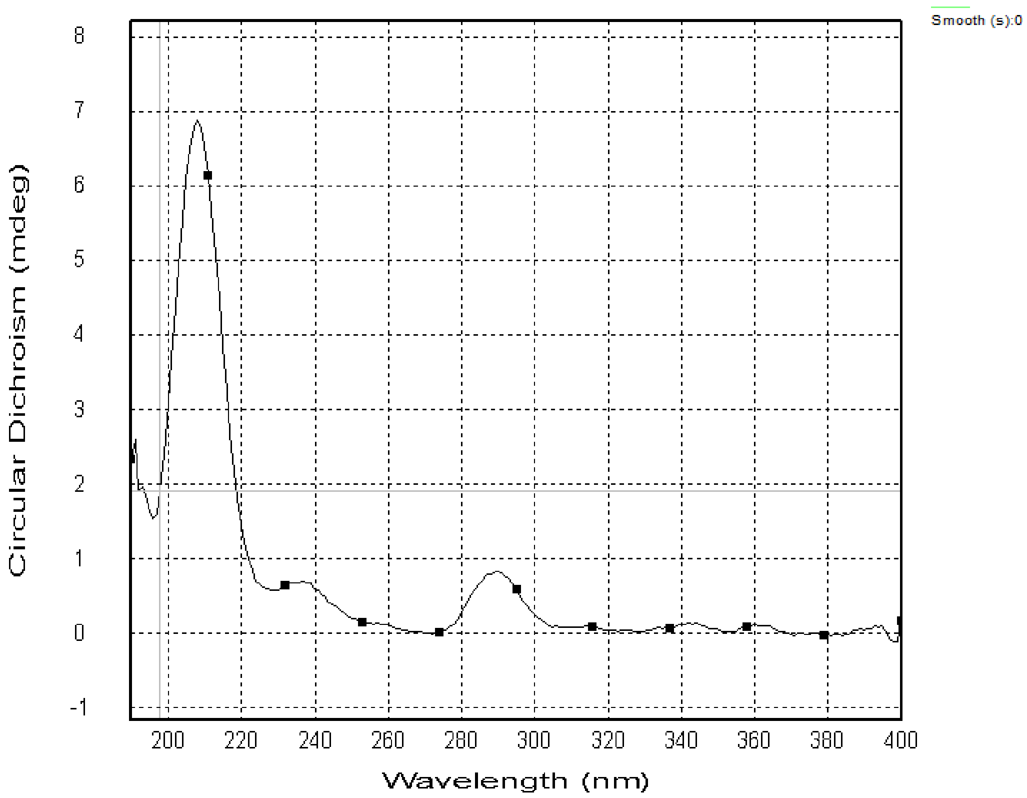


Figure S17. ECD spectrum of **2**

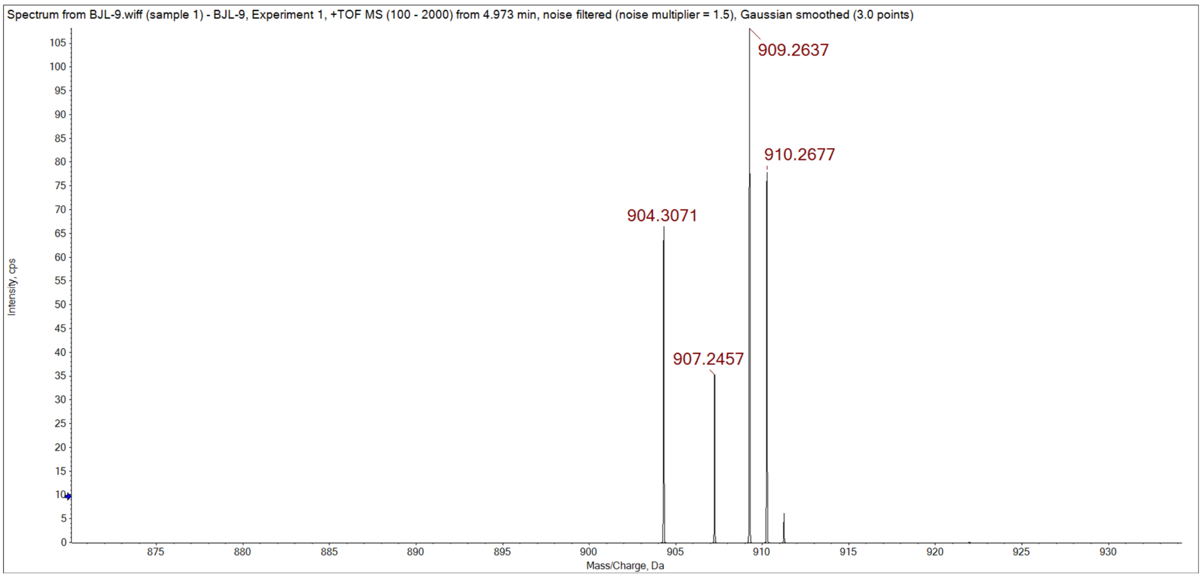


Figure S18. HR-ESI-MS spectrum of **2**

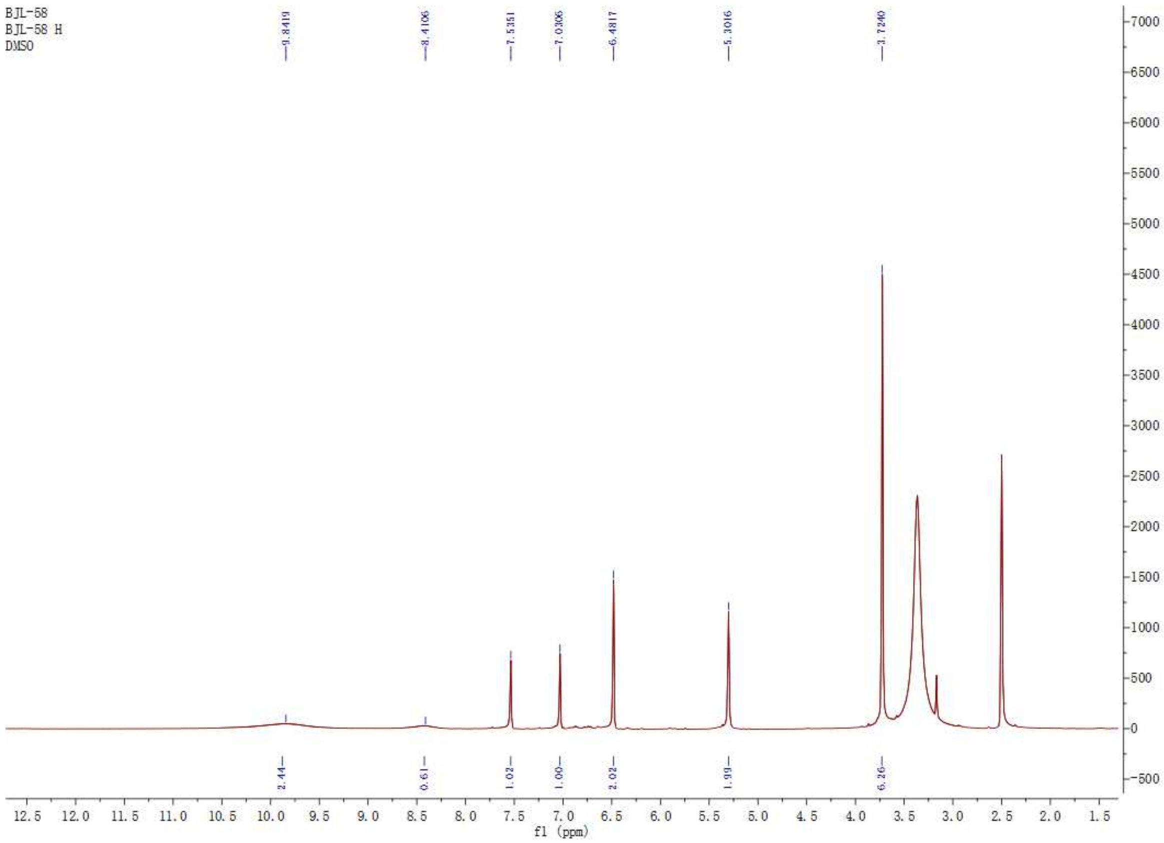


Figure S19. $^{1}H$ NMR spectrum of **3** (500 MHz, DMSO-*d*6)

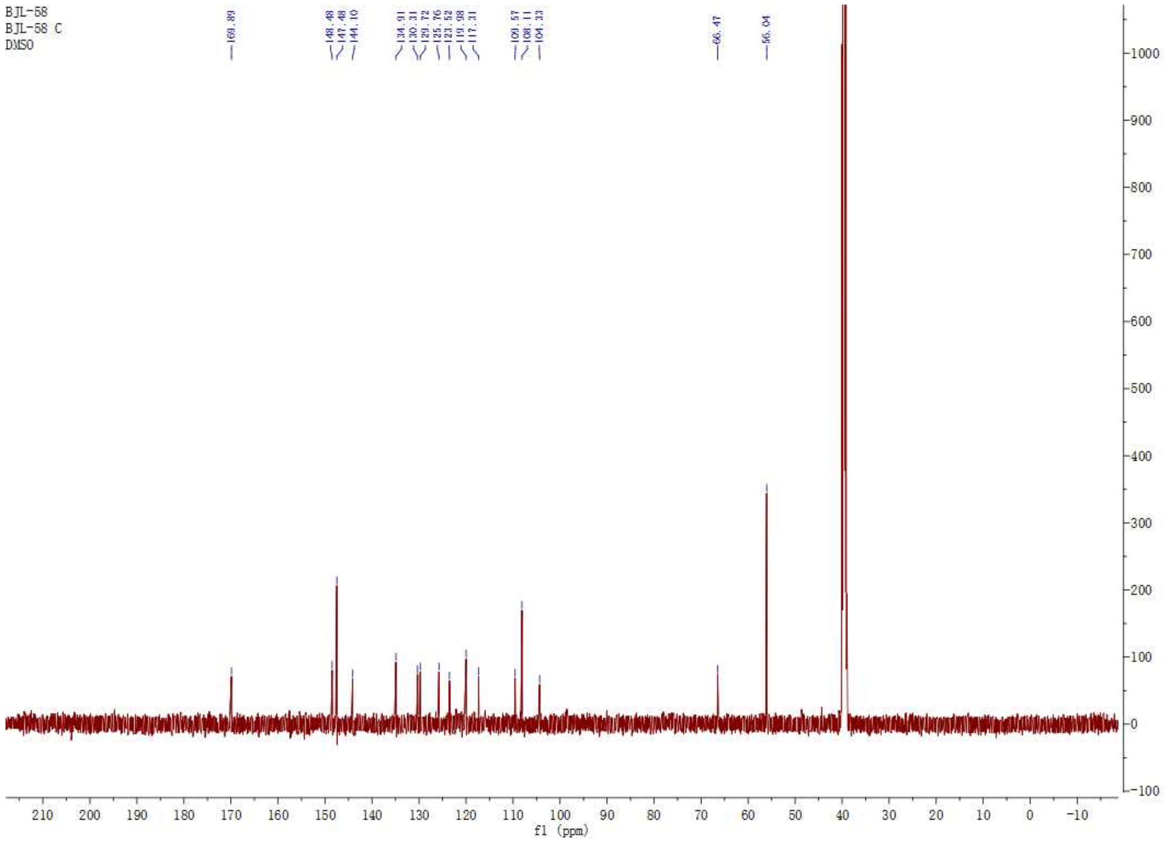


Figure S20. $^{13}C$ NMR spectrum of **3** (125 MHz, DMSO-*d*6)

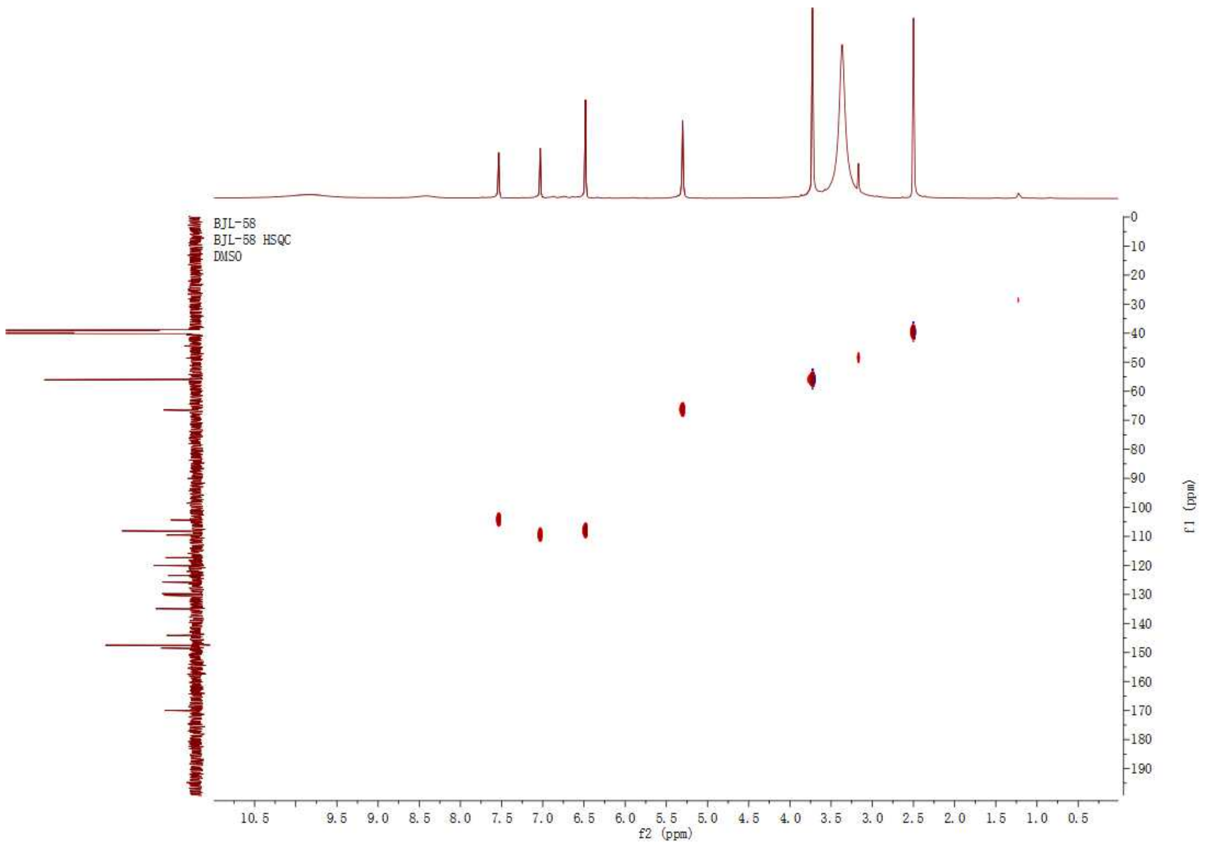


Figure S21. HSQC spectrum of **3**

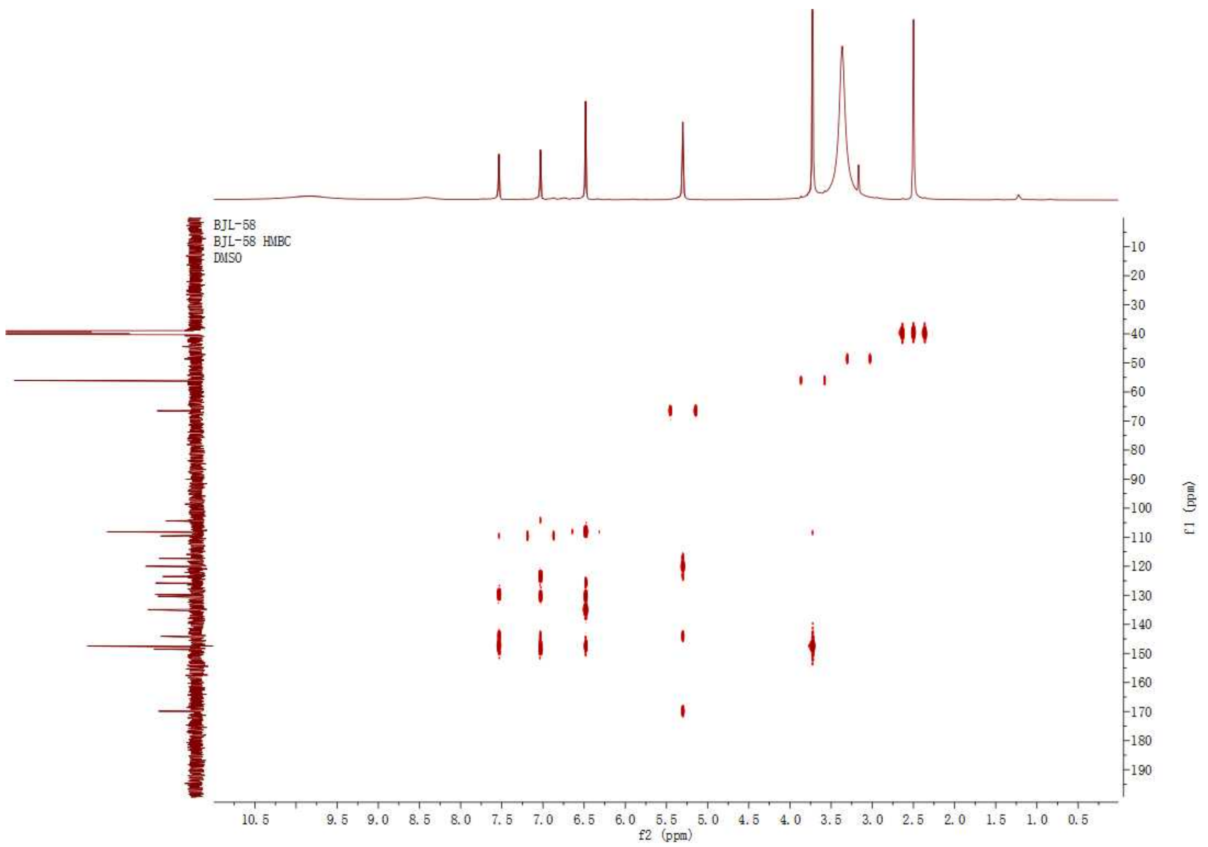

Figure S22. HMBC spectrum of **3**

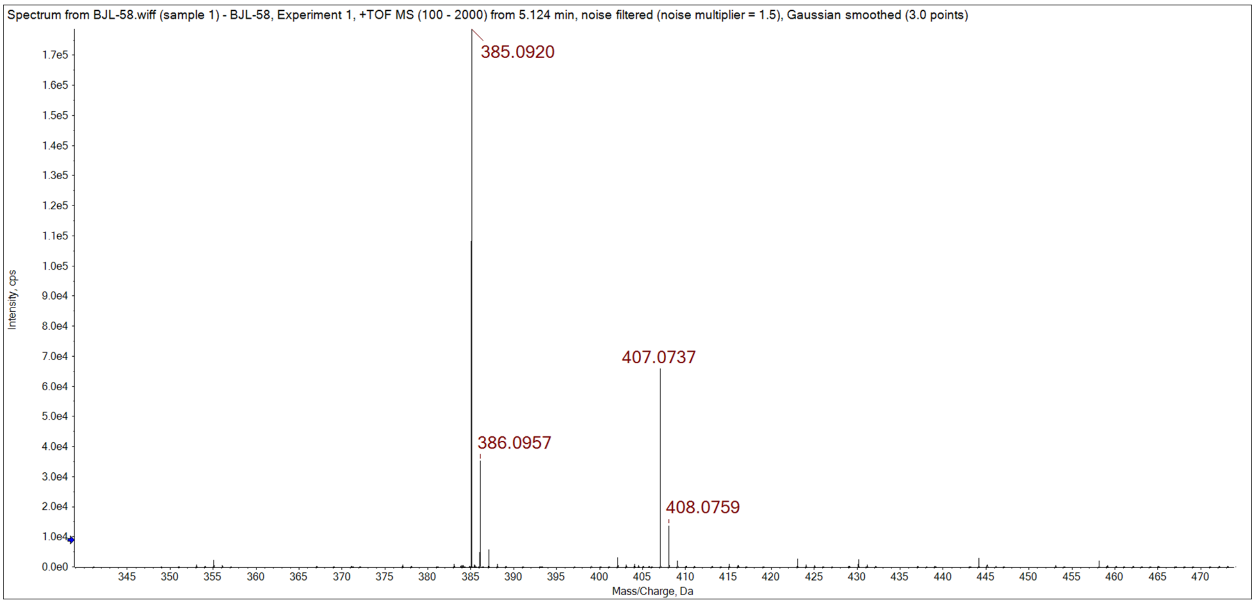


Figure S23. HR-ESI-MS spectrum of **3**

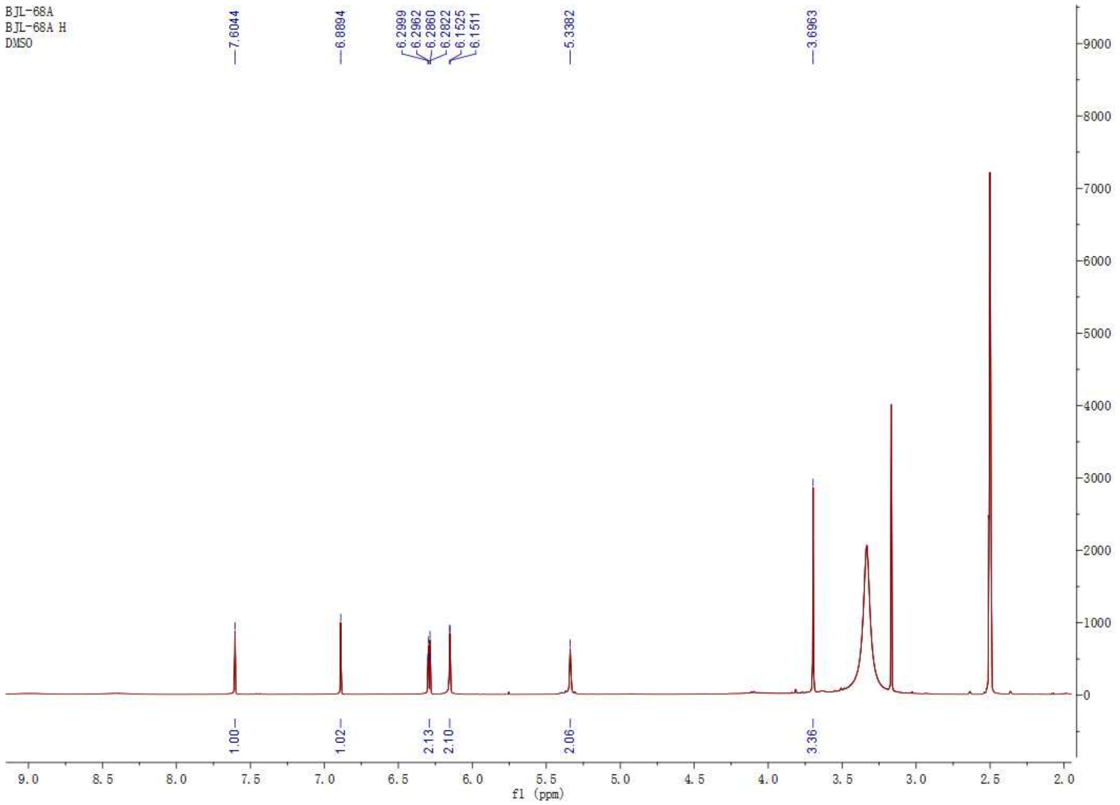


Figure S24. $^{1}$H NMR spectrum of **4** (500 MHz, DMSO-*d6*)

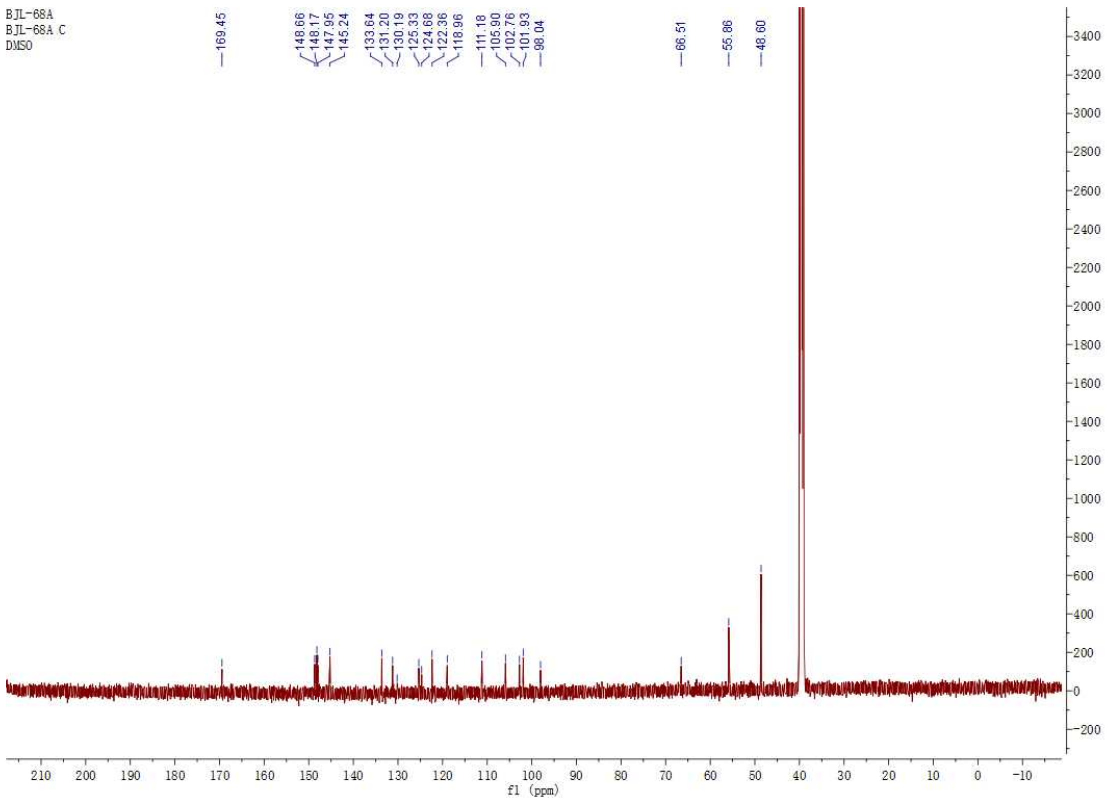


Figure S25. $^{13}$C NMR spectrum of **4** (125 MHz, DMSO-*d*6)

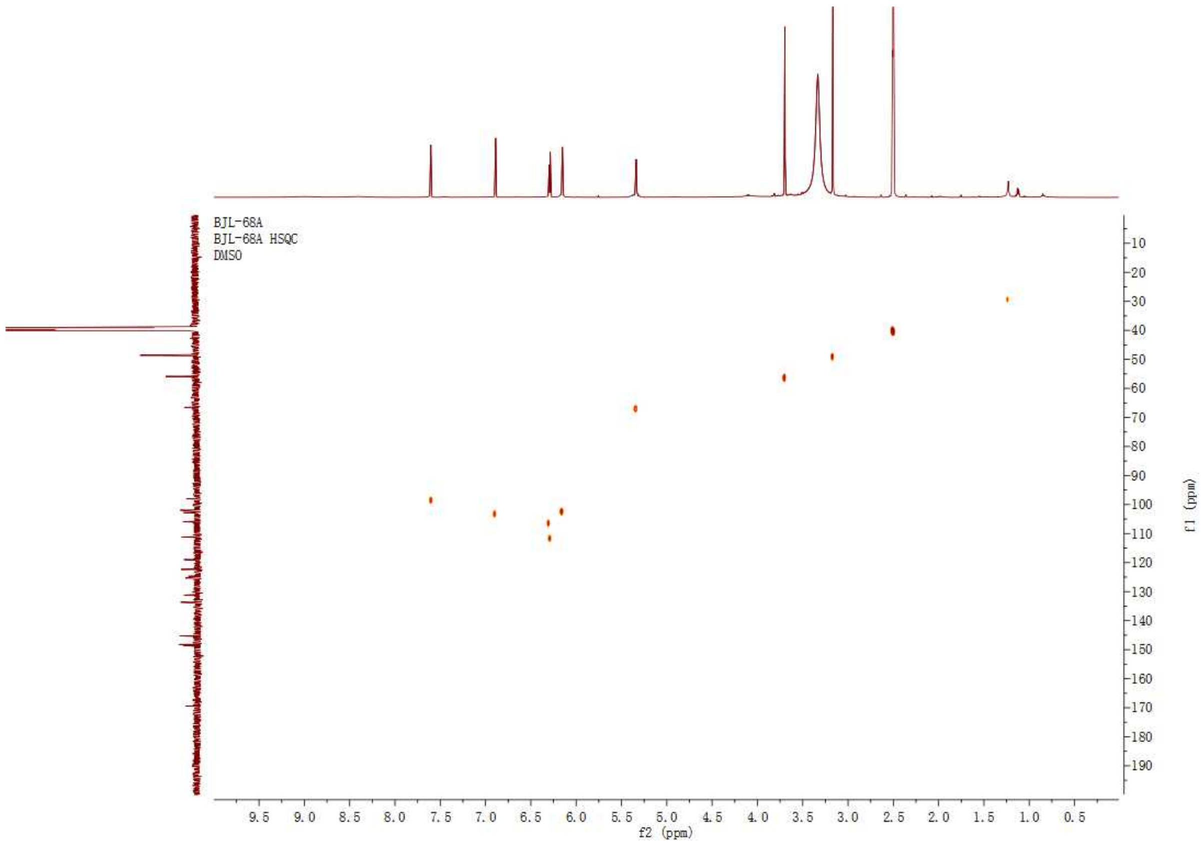


Figure S26. HSQC spectrum of **4**

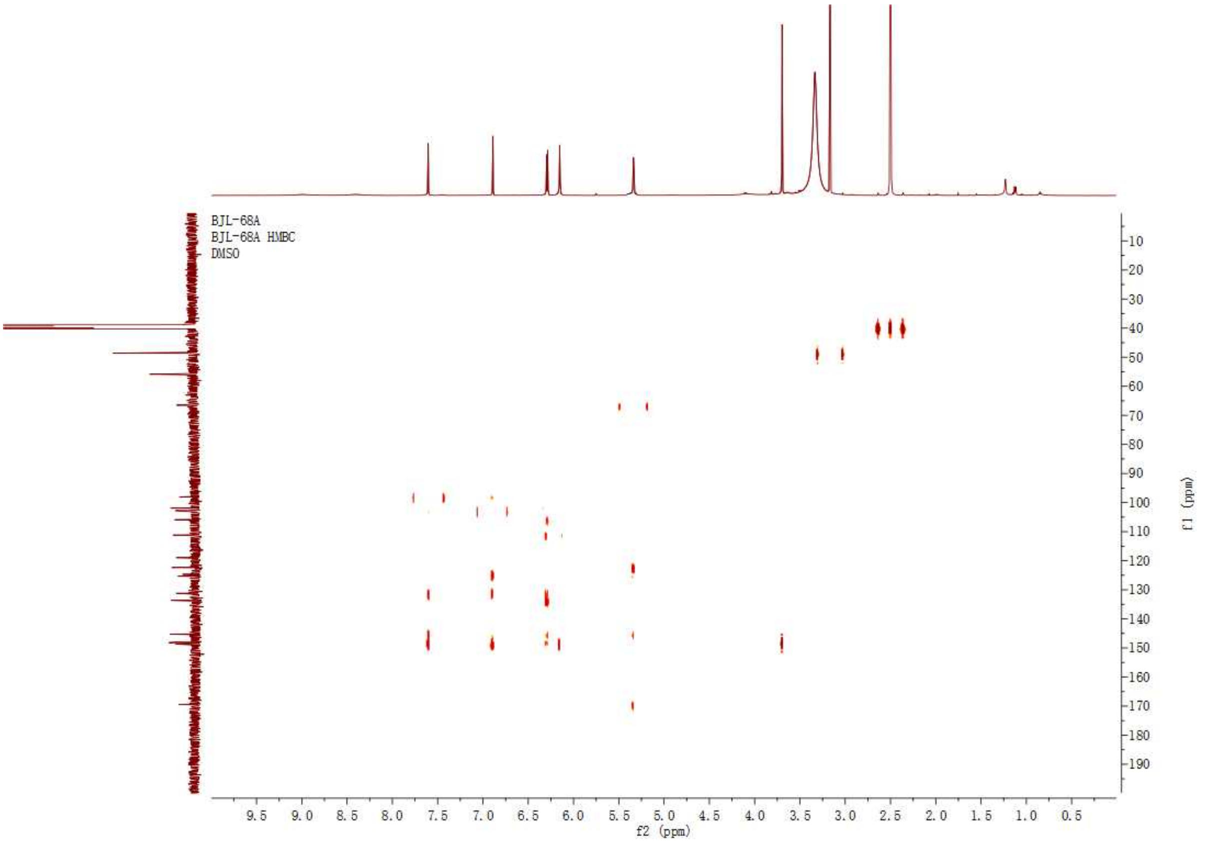


Figure S27. HMBC spectrum of **4**

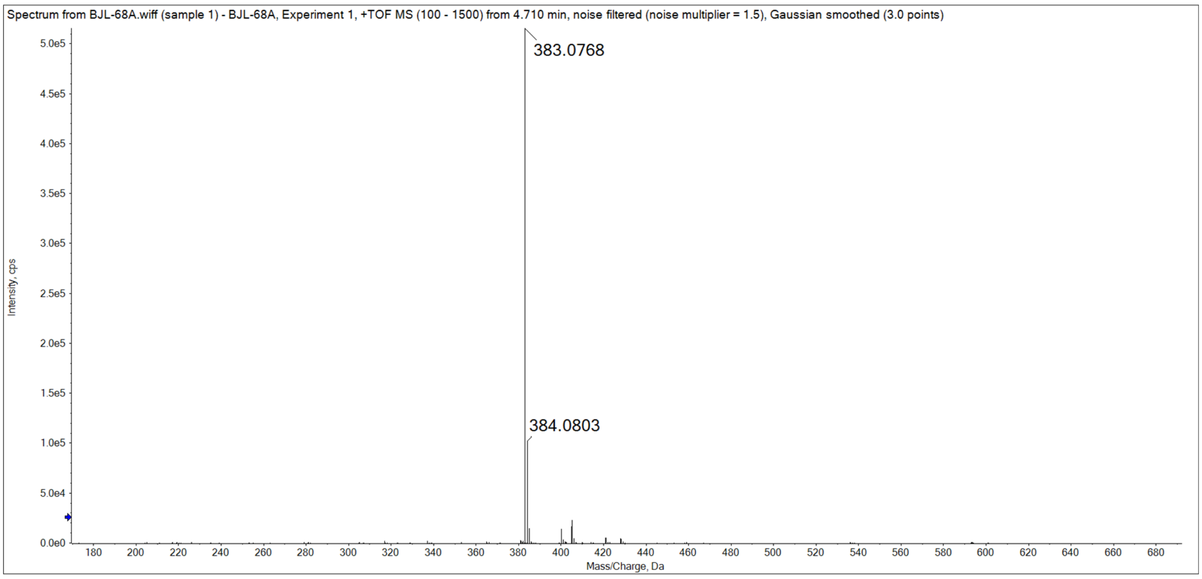


Figure S28. HR-ESI-MS spectrum of **4**
